# Retinal ganglion cells encode the direction of motion outside their classical receptive field

**DOI:** 10.1073/pnas.2415223122

**Published:** 2024-12-30

**Authors:** Serena Riccitelli, Hadar Yaakov, Alina S. Heukamp, Lea Ankri, Michal Rivlin-Etzion

**Affiliations:** ^a^Department of Brain Sciences, Weizmann Institute of Science, Rehovot 7610001, Israel

**Keywords:** extraclassical receptive field, direction selectivity, retinal ganglion cells, lateral geniculate nucleus, desensitization

## Abstract

Retinal ganglion cells (RGCs) typically respond to light stimulation over their spatially restricted receptive field. Using large-scale recordings in the mouse retina, we show that a subset of non- direction-selective (DS) RGCs exhibit asymmetric activity, selective to motion direction, in response to a stimulus crossing an area far beyond the classic receptive field. The extraclassical response arises via inputs from an asymmetric distal zone and is enhanced by desensitization mechanisms and an inherent DS component, creating a network of neurons responding to motion toward the optic disc. Pharmacological manipulations revealed the necessity of glycinergic amacrine cells for this response. Using in vivo recordings, we identified similar extraclassical responses in lateral geniculate nucleus neurons, suggesting such non conventional DS information is transferred to downstream structures. Our results suggest a complex integration of motion direction processing across the visual field, which arises beyond the classical receptive field boundaries.

## Significance

This study reveals that a subset of non- direction-selective retinal ganglion cells (RGCs) in the mouse retina exhibit asymmetric activity in response to moving stimuli from beyond their traditionally defined receptive field. This non conventional information is relayed to higher visual centers. These findings suggest that RGCs encode distinct features in their classical and extraclassical receptive fields and offer insights into motion processing in the visual system.

The receptive field (RF) of a cell is defined as the region where a stimulus elicits a neuronal response (1, 2). Retinal ganglion cells (RGCs), the retina’s output neurons, display a characteristic center-surround RF organization: ON RGCs are excited by light increments in the center but inhibited by increments in the surround (3, 4). OFF RGCs, excited by light decrements, display a similar center-surround antagonism. In the classical description, the center RF aligns with the cell’s dendritic field, while the inhibitory surround extends beyond, up to several hundred micrometers (5–9).

Decades ago, it was demonstrated that the activity of RGCs could be modulated by stimuli in the periphery, well beyond the classical RF, even over 1 mm away. This “periphery effect”, or extraclassical RF, can modulate RGC activity when combined with RF center stimulation (10–15). Through this effect, an RGC becomes sensitive to both the local stimulus and the context in which the stimulus is embedded.

While periphery effects were typically revealed by presenting grating patterns over large areas of the visual field, recent studies using single bars revealed that an RGC can respond differently to an object based on whether it is presented in its RF center or a distal area (16, 17). These findings suggested that RGCs can simultaneously encode distinct features in the classical and extraclassical RFs. Here, we investigated whether the extraclassical RF contributes to the retina’s well-known computation of motion direction. The direction of motion is locally encoded by direction-selective (DS) RGCs (DSGCs), which respond selectively to motion in the preferred direction within their RF (18–21). Other RGC types with asymmetric dendrites have been reported to exhibit DS responses under specific stimulus conditions, alongside maintaining robust encoding of different visual features (22, 23). Here, we show that a subset of non-DSGCs can generate a form of DS response to a faraway stimulus moving outside their classical RF.

We investigate the mechanisms underlying this asymmetric extraclassical response and demonstrate that it is not only spatially asymmetric but also selective to the direction of motion. We then demonstrate it also occurs in lateral geniculate nucleus (LGN) neurons. Since this response arises from a moving object well before it enters the classical RF, we speculate that this non conventional DS information may predict object trajectories, potentially compensating for delays in visual processing (e.g., due to phototransduction).

## Results

### RGCs Respond to Objects Moving Toward the Optic Disc Outside Their Classical RF.

**Fig. 1.**
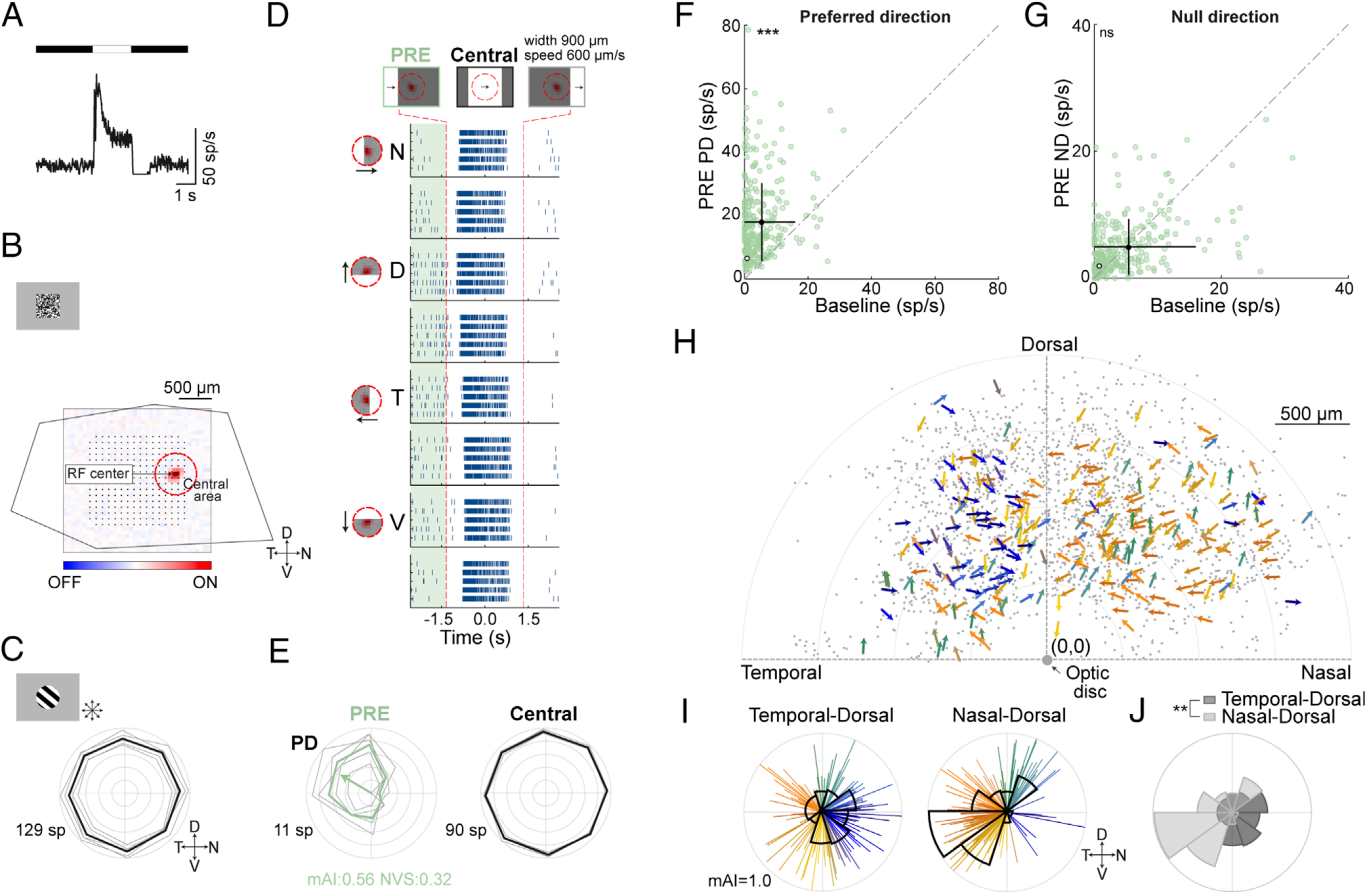
RGCs display asymmetric responses to moving stimuli in their extraclassical RF. A-D. Responses of an example ON RGC to various stimuli. (*A*) Peristimulus time histogram (PSTH, 5 repetitions, 10 ms bin) to a full-field spot stimulus. (*B*) Spatial RF from white-noise stimulation. Red and blue indicate ON- and OFF-responses. Black dots mark MEA electrodes. The red circle outlines a fixed “Central area” (350 μm radius) around the RF center. Retinal edges overlaid. (*C*) Polar plot for moving gratings. The bold line represents mean spike count over 4 s duration; thin lines denote single repetitions. (*D*) Raster plots for a bar moving in 8 directions (4 denoted on the *Left*). Each line is a trial. Bar’s position relative to the RGC’s RF center is illustrated above for different time points. Dashed red lines mark times when the bar’s leading/trailing edge enters/exits the Central area in (*B*). Shaded light green delineates PRE responses. t = 0 s corresponds to the time when the bar center is aligned with the RF center. (*E*) Polar plots calculated before the bar enters the Central area (PRE, shaded area in *D*) and while it crosses the Central area (black). Notations and coordinates as in (*C*). The arrow points to the preferred direction, with length representing the mAI (outermost radius equals 1). (*F*) Population data (272 cells, 15 experiments) showing the mean firing rate evoked by the bar moving in the preferred direction of the PRE response vs. baseline firing rate, with the example in *A*–*E* marked (white circle). (*G*) Same as *F*, except for the null direction of the PRE response. (*H*) Overlaid RGCs (2207 cells, gray dots represent the location of recorded RF centers) from both right and left dorsal retinas in the retinal space, with optic disc at (0,0). Arrows indicate the preferred directions of PRE RGCs. (*I*) Polar plots of preferred directions from temporal-dorsal (119 cells) and nasal-dorsal (141 cells) regions. Circular histograms are overlaid. (*J*) Direction preference circular histograms of PRE RGCs from *I*. Abbreviations: D, dorsal, N, nasal, V, ventral, T, temporal, indicating retinal coordinates; PD, preferred direction; ND, null direction; sp, spikes; mAI, motion asymmetry index, NVS, normalized vector sum. (*F–G*) ***: *P <* 0.001, ns, not statistically significant, two-sided Wilcoxon signed-rank test. (*J*) **: *P <* 0.01, Kuiper two-sample test.

We recorded the light-evoked spiking activity of RGCs from isolated mouse dorsal retinas using a multielectrode array (MEA). Although a portion of the cells may represent displaced spiking amacrine cells (24), we refer to all recorded units as RGCs. We presented the retinas with various visual stimuli, including a full-field spot to determine cells’ polarity preference (ON, OFF, or ON–OFF), spatiotemporal white-noise to locate the cells’ RF and estimate its size, as well as moving gratings and bars (Fig. 1 *A–D*). The bar (white on a black background, 900 μm wide) moved at 600 μm/s in 8 directions across the retina. For each RGC, we estimated the time of the bar’s leading edge entry into the RF and the trailing edge exit. As expected, cells responded robustly as the bar crossed their RFs (*SI Appendix*, Fig. S1).

Interestingly, in a subset of RGCs, the moving stimulus evoked spiking activity while crossing an area outside their classical RF before the leading edge entered it (Fig. 1*D* and *SI Appendix*, Fig. S2 *A* and *B*). Defining a circular ring around the classical RF (*SI Appendix*, Fig. S2*B*), we observed asymmetric responses when the bar approached the RF from one direction (referred to as the preferred direction, i.e., motion direction that triggers a response when the stimulus moves through an area beyond the classical RF) but not from the opposite direction (referred to as the null direction, i.e., when the stimulus crosses an area beyond the classical RF, but from the opposite side) (Fig. 1*E*). These responses, emerging before the bar entered the classical RF, are termed PRE responses. We considered a response as a spatially asymmetric PRE response when the motion asymmetry index (mAI) exceeded 0.3, the normalized vectorial summation (NVS) exceeded 0.15, the spike count crossed a minimal threshold per repetition, and it passed a permutation shuffling test (*Materials and Methods*). Below, we investigate the nature and origin of this asymmetric extraclassical response, demonstrating it is not only spatially asymmetric but often DS.

To ensure that PRE responses emerged in the extraclassical RF, we considered only those responses that occurred before the bar’s leading edge entered a predefined Central area, which extended 350 μm from the RF center (*Materials and Methods*). Additionally, we included only cells with RF centers located at least 450 μm away from the nearest retinal edge (referred to as Distance_min_). This criterion guaranteed that we considered RGCs, which extraclassical RFs extend within the retinal tissue (*SI Appendix*, Fig. S2*C*). Overall, 12.7 ± 2.0% (mean ± SEM) of the RGCs exhibited a PRE response. We explored the spatial extent of the region that contributed to the PRE response by changing the Central area size. The portion of PRE RGCs was stable for radii of 250 and 350 μm but decreased for larger Central areas, indicating the phenomenon is spatially confined (*SI Appendix*, Fig. S2*D*). Hereafter, we set the Central area radius to 350 μm, adjusting Distance_min_ for each RGC.

The PRE response may result from increased activity in the preferred direction or reduced activity in the null direction. To resolve this, we calculated the mean firing rate of PRE responses during preferred and null motion and compared them to the baseline firing rate (*Materials and Methods*). Our results reveal that the PRE response primarily derives from enhanced activity in the preferred direction motion. However, in a subset of cells, we observed reduced activity in the null direction, suggesting that inhibition may also contribute to the asymmetric response in these cells (Fig. 1 *F* and *G* and *SI Appendix*, Fig. S2*E*).

The preferred directions of PRE responses pooled across retinas did not significantly depart from a uniform distribution (Rao’s spacing test). However, aligning all recorded retinas to the optic disc (Fig. 1*H*) revealed differences between the temporal-dorsal and nasal-dorsal PRE RGCs (119 out of 881 and 141 out of 1089, representing 13.51 and 12.95% of the total recorded RGCs, respectively; *P <* 0.01, Kuiper two-sample test) (Fig. 1 *I* and *J*). This resulted in a directional bias in PRE responses toward the optic disc, with mean vectors of 333.5 ± 1.3° for temporal-dorsal and 182.8 ± 1.1° for nasal-dorsal populations (mean vector ± standard angular deviation), respectively.

Additional experiments and analyses confirmed the reliability and robustness of our results. First, we confirmed that the extraclassical RF responses were not due to spike sorting errors, ruling out the possibility that the phenomenon we describe here is due to the grouping of different cells with spatially offset RF (Supporting Information text and *SI Appendix*, Fig. S1). Second, we ruled out light aberration as a factor (Supporting Information text and *SI Appendix*, Figs. S3 and S4). These results validate that PRE responses are asymmetric and vary in their preferred directions according to retinal topography. Thus, PRE responses carry information about faraway motion direction, specifically from the periphery to the retina’s center.

### Asymmetric PRE Responses Primarily Emerge in Non-DS ON Sustained RGCs.

The relatively high proportion of PRE RGCs in our recordings suggests this group comprises multiple cell types. As the example in Fig. 1, asymmetric PRE responses tended to emerge in non-DSGCs (*P* <0.05, Fisher exact test; *SI Appendix*, Fig. S5*A*). We then characterized PRE RGCs by their response to full-field spots and found that PRE responses were predominantly displayed by ON RGCs (179 out of 272; *p <* 0.001, χ^2^ test; *SI Appendix*, Fig. S5 *B–E*), which presented larger RFs than average (*P <* 0.001, Kruskal–Wallis test with Bonferroni post hoc correction for multiple comparisons; *SI Appendix*, Fig. S5*F*). Clustering analysis suggests that ON PRE RGCs fall into five groups, with a dominance of sustained responses (*SI Appendix*, Fig. S5 *E*, *G* and *H*). Referring to the RGC typology project (rgctypes.org) (25) and response profiles, we suggest that the ON PRE RGCs include types like ONα (i.e., M4) (26), M2 (27, 28) and PixON RGCs (29), which correspond to 8w, 9w, and 9n RGCs in the EyeWire project (30). Since PRE responses were predominantly displayed by ON RGCs, we confined subsequent analyses to ON PRE RGCs.

### Flashed Static Bars Reveal the Existence of a Distal “Activation Zone”.

**Fig. 2.**
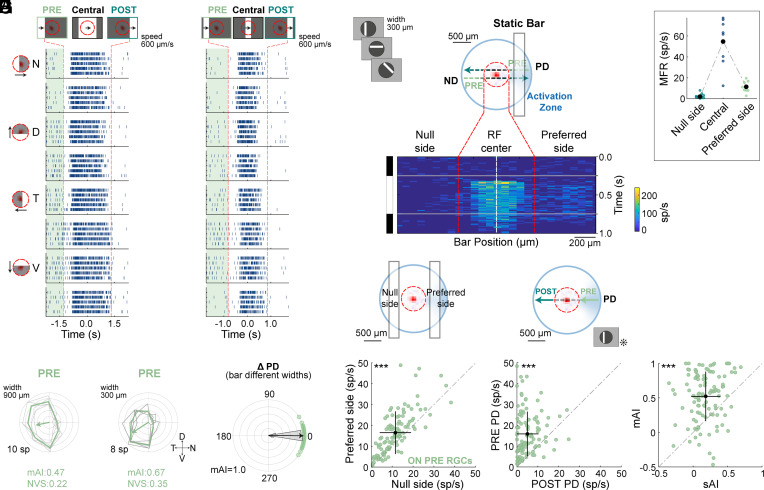
Static bars reveal an asymmetric extraclassical RF in ON PRE RGCs. (*A* and *B*) As in Fig. 1*D* for an example ON PRE RGC in response to moving bars 900 μm (*A*) and 300 μm (*B*) wide. (*C*) Polar plots as in Fig. 1*E* for 900 μm (*Left*) and 300 μm (*Right*) bars. (*D*) Polar plot showing ΔPD for PRE RGCs responding to 900 vs. 300 μm moving bars (108 cells, 9 experiments, green circles). Circular histogram is overlaid; the arrow indicates mean vector. (*E*) *Top*: Static bar stimulus illustration, activation zone asymmetrically located relative to the classical RF. Analysis focused on static bars oriented orthogonally to the preferred-null axis for each cell. *Bottom*: Average firing rate (4 repetitions, color map on the *Right*) of the example RGC in response to a 500 ms static bar (stimulus on the *Left*). *X*-axis: bar’s position relative to RF center (dashed white line); *Y*-axis: time. Dashed red lines indicate the Central area. *Inset*: Mean firing rate for all bar positions flashed in the null side, Central area, and preferred side. The averages across these positions are denoted in black. (*F*) *Top*: Illustration of the responses analyzed with respect to the classical RF. Average responses (as in *E*, *Inset*) in the preferred vs. null side for all ON PRE RGCs (108 cells, 9 experiments). (*G*) Same as *F*, except for a bar moving in the preferred direction of the asymmetric PRE response. (*H*) Comparison of asymmetric index (AI) for moving (mAI) vs. static (sAI) bars. In *F–H*, the mean ± STD is shown for both axes, black lines. Abbreviations as in Fig. 1. (*F*–*H*) ***P < 0.001 according to the two-sided Wilcoxon signed-rank test.

A plausible explanation for the appearance of the PRE response is an asymmetric input from a distal area outside the classical RF, located on the RGC’s preferred side, which corresponds to the origin of the PRE preferred direction. We term this asymmetric extraclassical RF the activation zone. To test this, we flashed stationary bars for 500 ms at different orientations and locations in a pseudorandom order. These static bars, 300 μm wide, offered higher spatial resolution than the original 900 μm moving bar. For comparison, we also presented moving bars of the same size. ON PRE RGCs displayed similar directional preferences for the 900 and the 300 μm moving bars (Fig. 2 *A–D*), with an average preferred direction difference (ΔPD) of − 0.36 ± 9.04° (mean vector ± standard angular deviation, n = 108), showing no deviation from 0° (one-sample test for the mean angle) (Fig. 2*D*).

We compared the activity in response to the appearance of the static flashed bars on the distal preferred side (the hypothesized activation zone) and on the opposite, null side, using bars oriented orthogonally to the PRE preferred direction (Fig. 2*E* and *Materials and Methods*). Similar to the example RGC, on average, ON PRE RGCs showed a significantly higher firing rate for bars flashed on the preferred side compared to the null side (Fig. 2*F*, 16.67 ± 0.98 vs. 11.61 ± 0.72 spikes/s, *P <* 0.001, two-sided Wilcoxon signed-rank test; mean ± SEM, n = 108). To extend this analysis to moving bars, we compared the average firing rates on the preferred and null sides as the bar moved in the PRE response’s preferred direction. For this purpose, we defined the POST response as the activity occurring after the bar’s trailing edge left the RGC’s Central area (*SI Appendix*, Fig. S2*A*). In line with the static bars results, ON PRE RGCs displayed more significant activity in the preferred side (PRE PD) than the null side (POST PD) for the 300 μm moving bar (Fig. 2*G*, 15.96 ± 1.02 vs. 5.07 ± 0.51 spikes/s, *P <* 0.001, two-sided Wilcoxon signed-rank test; mean ± SEM). To quantify the imbalance in activation between the preferred and null sides, we compared the asymmetric index for motion vs. static bars (mAI vs. sAI) (*Materials and Methods*). Results showed significantly higher AI values for moving bars, indicating that motion in the activation zone elicits more robust responses in ON PRE RGCs (Fig. 2*H*, mAI 0.52 ± 0.03 vs. sAI 0.18 ± 0.02, *P* < 0.001, two-sided Wilcoxon signed-rank test; mean ± SEM).

### The Activation Zone Predicts POST Responses Tuned to the Opposite Direction.

**Fig. 3.**
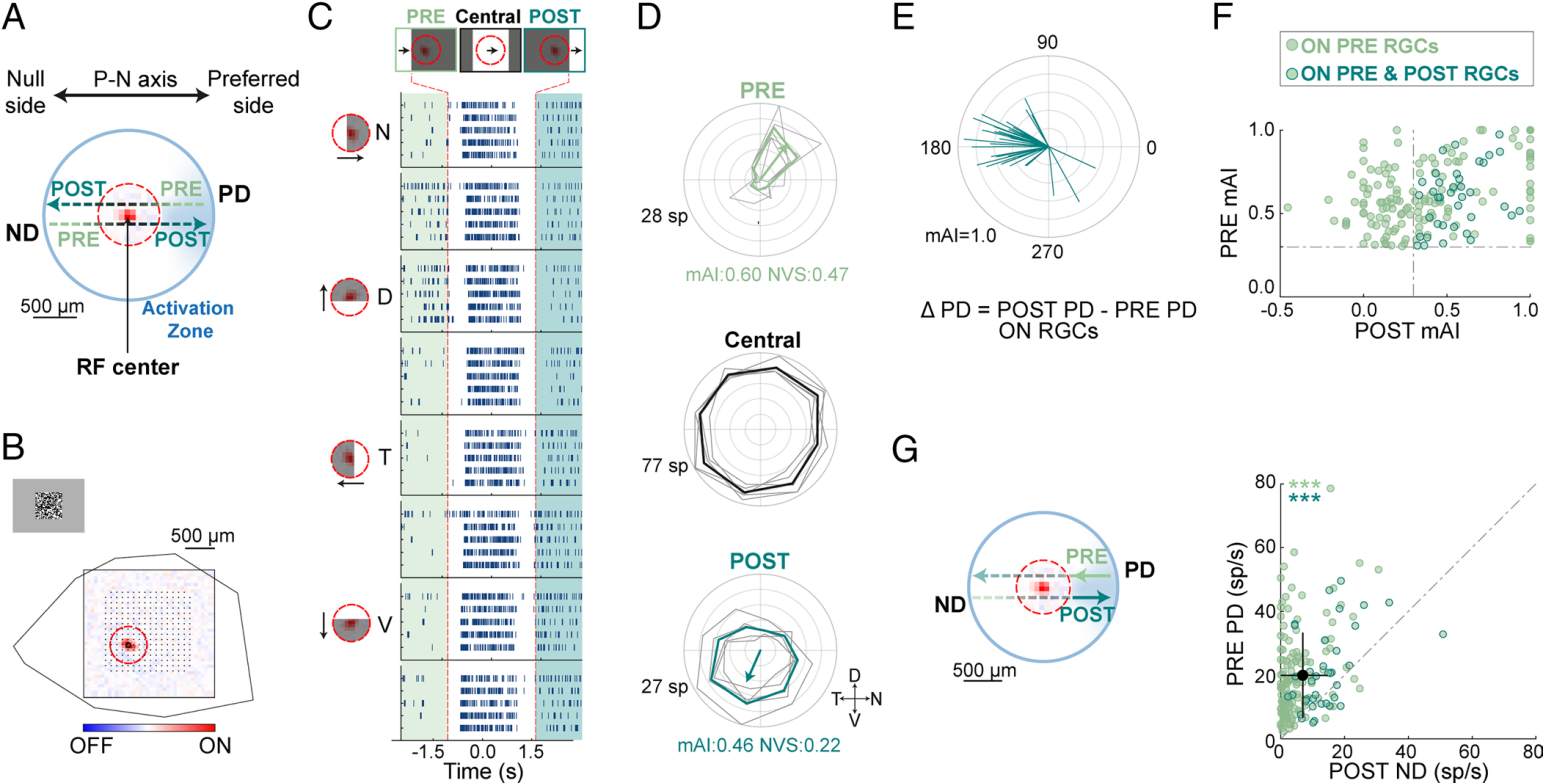
A fifth of ON PRE RGCs reveal an oppositely tuned asymmetric POST response. (*A*) Sketch of the asymmetric activation zone. (*B*) Spatial RF from white-noise stimulation. Notations as in Fig. 1*B*. (*C*) Same as in Fig. 1*D* for an example RGC, with shaded light- and dark-green areas delineating PRE and POST responses, respectively. (*D*) Polar plots of the example RGC’s responses to the moving bar calculated prior to its entrance into the Central area (PRE, light green), while the bar crosses the Central area (black) and when the trailing edge of the bar leaves the Central area (POST, dark green). Notations as in Fig. 1*E*. (*E*) Polar plot showing POST preferred directions for all ON PRE RGCs with asymmetric POST responses (37 cells, 15 experiments). PRE preferred direction aligned to 0°. Arrow length indicates POST mAI. (*F*). PRE and POST mAIs, identifying 37 of 179 ON PRE RGCs with asymmetric POST responses (dark green). (*G*) Population data (179 cells, 15 experiments) showing responses to a moving bar in the PRE PD vs. POST ND over the activation zone (*Left*: solid arrows depict the analyzed area). The preferred and null directions are relative to the PRE response. Mean ± STD is shown for both axes, black lines. Abbreviations as in Fig. 1. (*G*) ***: *P <* 0.001 according to the two-sided Wilcoxon signed-rank test.

Identifying an asymmetric activation zone suggested that ON PRE RGCs might also show POST responses tuned to the opposite direction of motion. This hypothesis arises because the bar crosses the same area when moving in the PRE preferred direction before reaching the Central area (leading edge response) and when moving in the opposite, PRE null direction (trailing edge response) after leaving the Central area (Fig. 3*A*). To test this, we calculated the directional tuning of POST responses.

As expected, some ON RGCs displayed oppositely tuned PRE and POST responses (Fig. 3 *B–E*; ΔPD = 176.83 ± 37.54°; n = 37 out of 179 ON PRE RGCs, mean vector ± standard angular deviation. The mean distribution of the ΔPD did not significantly differ from 180°, one-sample test for the mean angle). Yet, PRE and POST responses were imbalanced. First, only a fifth of ON PRE RGCs exhibited asymmetric POST responses. Although many PRE RGCs exhibited POST mAI values higher than 0.3, these responses were inconsistent with low spike counts (Fig. 3*F* and *Materials and Methods*). Second, comparing firing rates in the activation zone during PRE preferred direction (PRE PD) vs. the null direction (POST ND) revealed higher activity for PRE PD motion (Fig. 3*G* 19.95 ± 1.00 spikes/s in PRE PD vs. 6.88 ± 0.58 spikes/s in POST ND, *P <* 0.001, two-sided Wilcoxon signed-rank test; n = 179, mean ± SEM). Thus, while oppositely tuned PRE and POST responses exist in some cells, additional mechanisms reduce POST ND responses, generating an asymmetric extraclassical RF that encodes the direction of motion (i.e., imbalanced evoked responses when the moving stimulus traverses the activation zone in the two opposite directions).

### Desensitization Mechanisms Contribute to the Directional Responses in the Extraclassical RF.

We suspected that desensitization mechanisms (i.e., reduced RGC responsiveness to further stimulation) contribute to the imbalanced responses of ON PRE RGCs arising in the activation zone during preferred and null motion. When the bar moves in the PRE preferred direction, it encounters the activation zone before reaching the RGC’s RF. In the null direction, the bar first strongly activates the RGC via the classical RF, reducing its subsequent response in the activation zone. This creates a larger response when the stimulus moves in the PRE PD than in the POST ND. Based on this hypothesis, an RGC response in the extraclassical activation zone depends on prior stimulation.

**Fig. 4.**
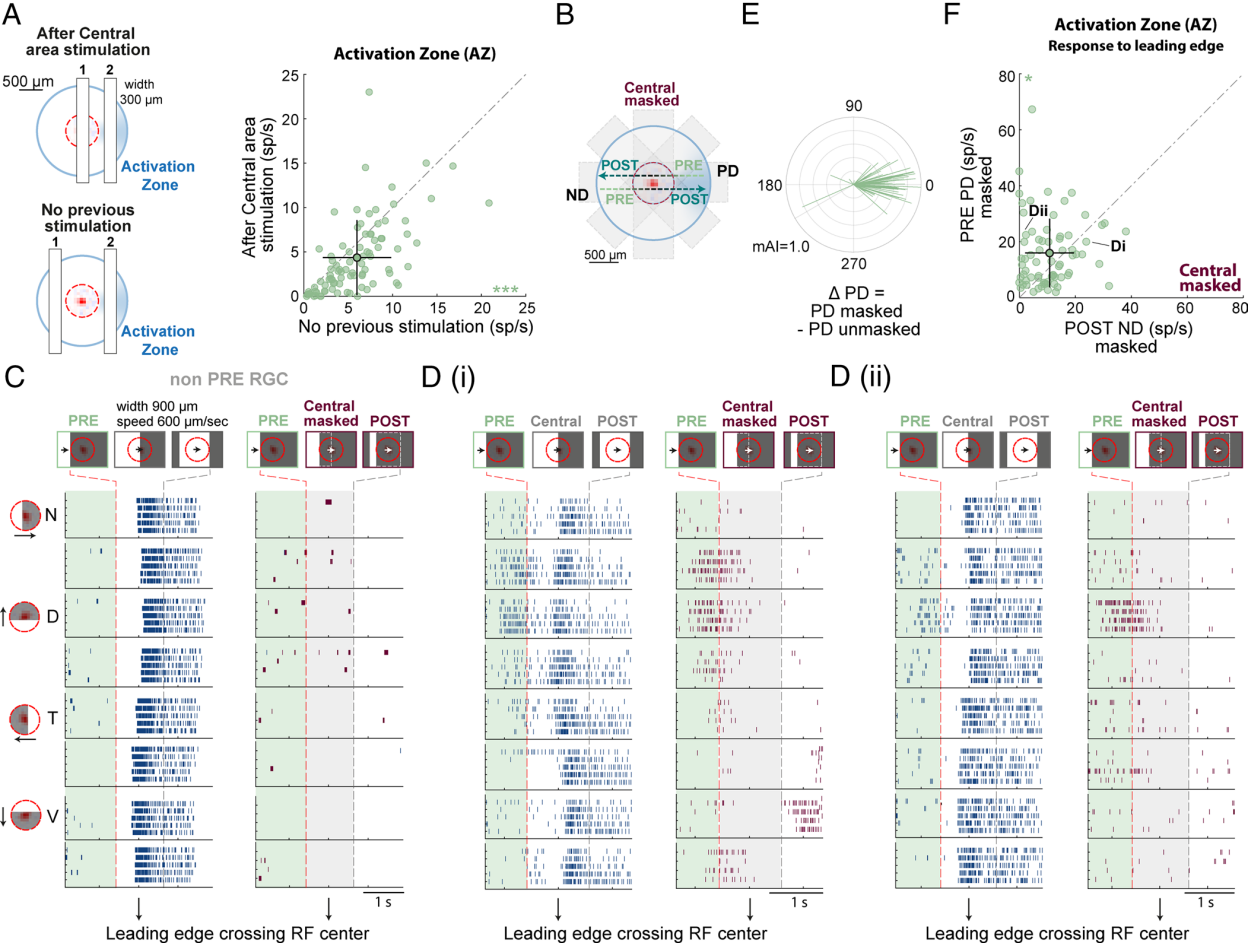
DS-like responses in the activation zone rely on desensitization mechanisms and an inherent DS component. (*A*) *Left*: Static bar presentations in the activation zone (2) were split according to previous stimulus location (1). *Right*: Comparison of the mean response to a static bar in the activation zone after Central area stimulation vs. no previous stimulation (87 ON PRE-only RGCs, 9 experiments). (*B*) Moving bar stimulus repeated while masking the Central area. Gray shades represent the masked area (width 700 μm) in each direction. (*C*). Raster plots of an ON non-PRE RGC in response to a bar moving in 8 directions, under unmasked (*Left*) and masked (*Right*) conditions. The location of the bar stimulus showed either over the full extent of the display or masked by an occlude is illustrated on top for different time points. Dashed red/gray lines indicate when the bar’s leading edge enters/exits the Central area. Shaded light green/gray delineates the PRE response/masked central area. Black arrow below depicts when the bar’s leading edge reaches the RF center. (*D*) (i&ii). As in *C*, for two ON PRE RGCs. (*E*) Polar plot of PRE preferred directions for ON PRE-only RGCs (70 cells, 5 experiments) after masking the central area. PRE preferred direction in the unmasked condition aligned to 0°. Arrow lengths represent the mAI in the masked condition. (*F*) Population responses to a moving bar in the PRE PD vs. POST ND over the activation zone in the masked condition. Note that both PRE PD and POST ND responses are responses to the bar’s leading edge over the activation zone. In *A*, *F*, Mean ± STD is shown for both axes, black lines. Abbreviations as in Fig. 1. (*F*) *: *P* < 0.05, (*A*) ***: *P <* 0.001 according to the two-sided Wilcoxon signed-rank test.

To validate this hypothesis, we focused on ON PRE RGCs that presented no asymmetric POST responses (asymmetric PRE-only) and examined their responses to the appearance of static flashed bars in their preferred side (i.e., the activation zone). Trials were grouped based on the location of the static bar in the previous trial. When the previous bar was presented in the Central area (Fig. 4*A*, “after Central area stimulation”), we expected a decreased response in the subsequent trial due to desensitization. Alternatively, we expected no decrease in the response when the previous bar was presented neither in the Central area nor in the activation zone (“no previous stimulation”). In line with our prediction, ON PRE RGCs significantly reduced their spiking activity to a stimulus flashed in the activation zone following Central area stimulation compared to trials with no previous stimulation (Fig. 4*A*, right; after Central area stimulation 4.38 ± 0.45 spikes/s vs. no previous stimulation 6.01 ± 0.41 spikes/s; *P <* 0.001, two-sided Wilcoxon signed-rank test; n = 87, mean ± SEM). Thus, while PRE responses originate from a distal activation zone, their amplitude is modulated by prior activation of the classical RF. Desensitization mechanisms reduce POST responses and contribute to generating an asymmetry with a directional preference that favors objects moving toward the RGC’s classical RF.

In a subset of experiments, we assessed how desensitization contributes to reduced POST responses by masking the Central area during moving bar stimulation. This isolation of the extraclassical RF responses prevented classical RF stimulation and subsequent desensitization, allowing a more precise comparison of PRE and POST responses (Fig. 4*B* and *SI Appendix*, Fig. S6 and *Materials and Methods*). As expected, the masking reduced Central area activity, with ~80% of ON RGCs not presenting an asymmetric PRE response decreasing their spiking activity by at least 95% (see example in Fig. 4*C*). Central masking did not affect the directional preference of ON PRE RGCs (Fig. 4 *D* and *E* ΔPD = 2.29 ± 30.45°; n = 70, mean vector±standard angular deviation. The mean distribution of the ΔPD did not significantly differ from 0°, one-sample test for the mean angle). Interestingly, in the presence of the central mask, ~50% of ON PRE RGCs showed more robust PRE responses, which also tended to be more prolonged (example in Fig. 4*D i* and *ii*). This suggests that, in the unmasked condition, the canonical surround inhibits RGC activity, halting PRE PD responses once the bar enters the RF. This mechanism separates classical and extraclassical responses into two distinct phases. We then compared mean firing rates evoked by the bar’s leading edge in the activation zone for PRE PD vs. POST ND in the masked condition. If desensitization alone underlies the imbalanced PRE PD and POST ND responses, we expect comparable activity in the activation zone in the two opposite motion directions. This was not the case: although we found cells that showed comparable PRE and POST responses (Fig. 4*D i*), in ~60% of the cases the activation zone presented DS-like characteristics (Fig. 4*D ii*), favoring PRE PD even when desensitization was prevented (Fig. 4*F*; PRE PD masked 15.98 ± 1.47 spikes/s vs. POST ND masked 10.82 ± 1.04 spikes/s; *P <* 0.05, two-sided Wilcoxon signed-rank test; n = 70, mean ± SEM). The imbalanced activity could not be solely explained by the mask's partial coverage of the activation zone, as the same mask was used when the bar was moving in opposite directions. Based on these findings, we conclude that asymmetric characteristics of the extraclassical responses in ON PRE RGCs are not merely a byproduct of the asymmetric activation zone and desensitization but imply an inherent DS component, i.e., a stronger response to the preferred direction of motion. This finding is further supported by the asymmetry between preferred and null side responses being more prominent for moving than for static stimuli (Fig. 2*H*).

We then used clustering analysis to determine whether specific ON PRE RGC types are associated with the DS characteristics of extraclassical responses. Cells were classified into clusters from *SI Appendix*, Fig. S5 based on their full-field spot responses (*SI Appendix*, Fig. S5*I*). Cluster 4 had the highest proportion of cells (75%) which showed an inherent DS component in the activation zone, significantly more than the other clusters (Fisher’s exact test, *P* = 0.0136). In contrast, Clusters 3 and 5 exhibited only a modest or no difference between PRE PD and POST ND responses, suggesting their responses primarily rely on desensitization.

### PRE Responses Remain Directionally Tuned Across a Broad Range of Speeds.

**Fig. 5.**
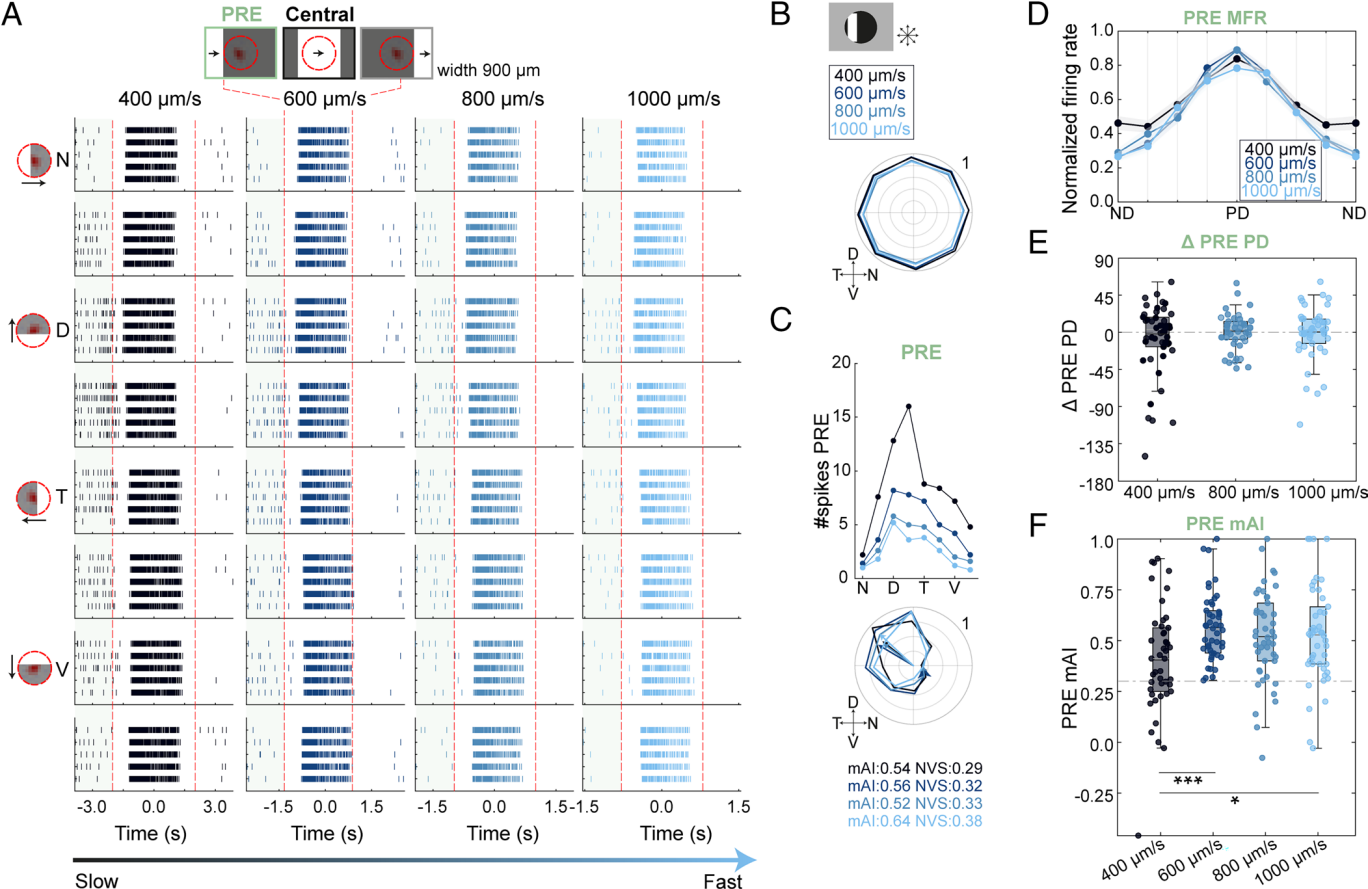
The PRE response is maintained across a range of speeds. (*A*) Raster plots of an example cell responding to bars moving at different speeds (denoted on *Top*). Notation as in Fig. 1*D*. (*B*) Polar plot of the example cell’s normalized mean responses in the Central area, color-coded by speed. (*C*) *Top*: Average PRE spike counts for the example cell, shown by direction and speed. Bottom: Polar plot of normalized responses. The arrows point to the preferred directions and their length represents the mAI value. Color-coding as in *B*. (*D*) Normalized mean±SEM PRE responses across all directions, aligned to the PRE PD at 600 μm/s (45 cells, 6 experiments), for each speed. (*E*) Box plot showing differences in PRE preferred directions at 600 μm/s vs. other speeds across RGCs. (*F*) Box plot of mAI values of PRE responses across speeds. Abbreviations as in Fig. 1. (*F*) *P < 0.05, ***: *P <* 0.001, according to Friedman’s test with Tukey–Kramer correction.

The asymmetric PRE response demonstrates that some RGCs can respond to a moving object before it enters their RF. Such responses may be beneficial to overcome a known processing delay due to phototransduction and synaptic transmission (31–33). To test whether PRE responses compensate for this delay during high-speed motion when the retinal delay is more prominent, we presented the retina with bars moving at 400, 600 (the original speed), 800, and 1,000 μm/s. Fig. 5 *A–C* depicts an example ON RGC that displayed consistent asymmetric PRE response over all tested speeds. Despite variations in spike count, the preferred direction, mAI, and tuning width of the PRE responses were relatively stable (Fig. 5*C*). To quantify the velocity preference of the population, PRE responses were aligned to the PRE preferred direction at 600 μm/s and normalized to the peak firing rate separately for each speed. Although the number of spikes elicited by moving bars decreased as speed increased, the population of ON RGCs revealed comparable asymmetric PRE responses across speeds (Fig. 5 *D* and *E*; ΔPD=−2.44 ± 39.53°, 0.21 ± 21.50° and −0.65 ± 29.85° for 400, 800, and 1,000 μm/s compared with the 600 μm/s; ns, nonparametric multisample test for equal medians; n = 45, mean vector ± standard angular deviation). mAI values only differed slightly at the slowest speed (Fig. 5*F*; 0.41 ± 0.04, 0.56 ± 0.03, 0.51 ± 0.03, and 0.52 ± 0.04 for 400, 600, 800, and 1,000 μm/s; *P <* 0.05 for the slowest speed vs. 600 and 1,000 μm/s, Friedman’s test with Tukey–Kramer correction; mean ± SEM). In line with this result, we also noticed a significant broadening (NVS) of the PRE response at the slowest speed (0.22 ± 0.02, 0.30 ± 0.02, 0.28 ± 0.02, and 0.31 ± 0.02 for 400, 600, 800, and 1,000 μm/s; *P <* 0.01 for the slowest speed vs. all, Friedman’s test with Tukey–Kramer correction; mean ± SEM).

### Glycinergic Amacrine Cells and Gap Junctions Contribute to PRE Responses.

**Fig. 6.**
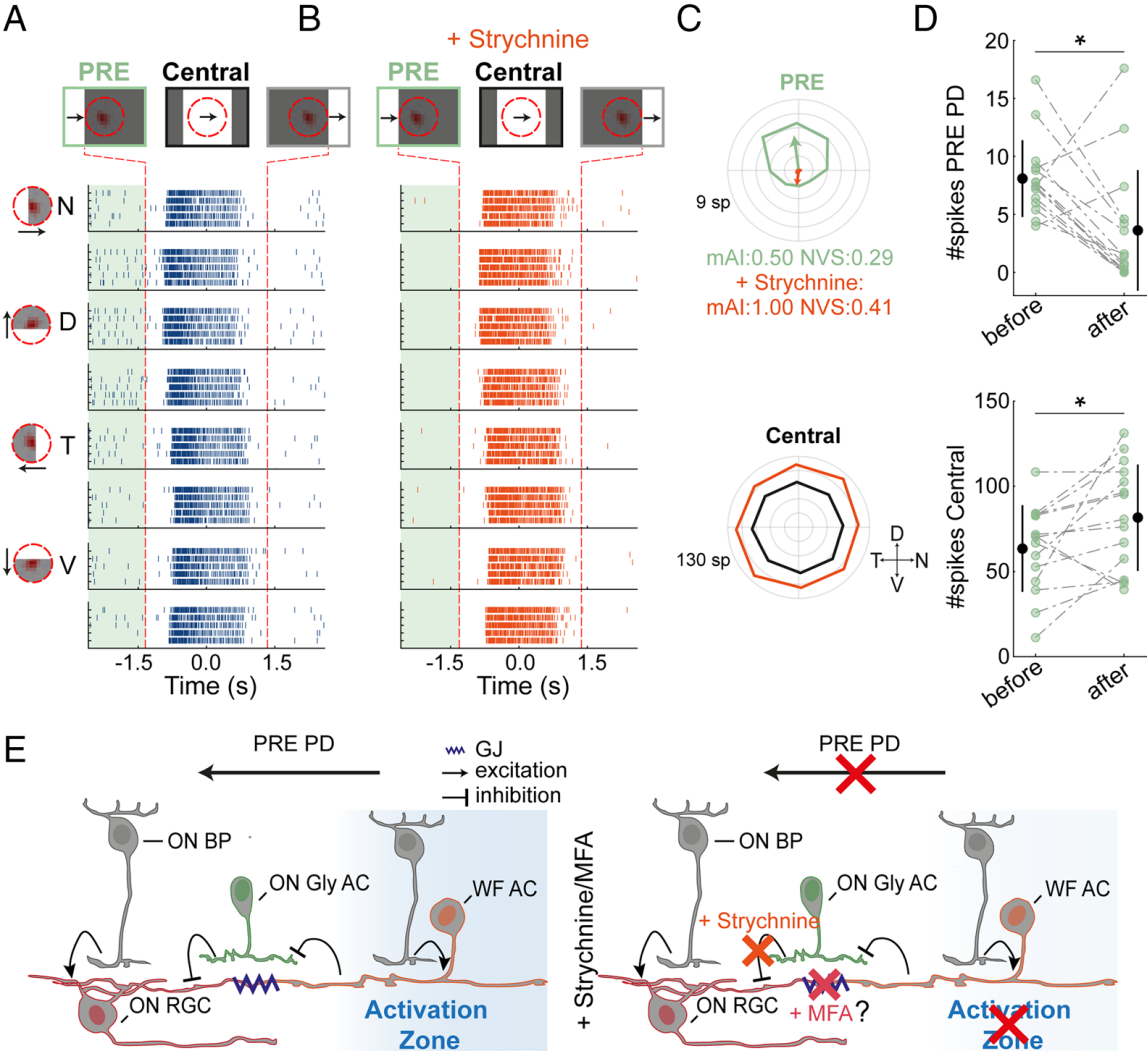
The extraclassical RF relies on glycinergic amacrine cells. (*A* and *B*) Raster plots of an example cell’s response to bars moving in 8 directions before (*A*) and after (*B*) strychnine application. Notations as in Fig. 1*D*. (*C*) Polar plots of RGC responses in the PRE (*Top*) and Central (*Bottom*) areas. The mean response is plotted before (light green/black) and after (orange) strychnine application. Notation as in Fig. 1*E*. (*D*) Spike counts in the PRE (*Top*) and Central area (*Bottom*) before and after strychnine application. Mean ± STD is shown, black lines. 15 cells, 4 experiments. (*E*) Simplest circuitry underlying asymmetric PRE responses in control (*Left*), and with strychnine or MFA (*Right*). See text for details. Abbreviations: BP, bipolar cell; ON Gly AC, ON Glycinergic amacrine cell; WF AC, wide-field amacrine cell; GJ, gap junction, PD, preferred direction. (*D*) *P < 0.05, according to the two-sided Wilcoxon signed-rank test.

To gain insight into the circuitry underlying asymmetric PRE responses, we explored the role of glycinergic amacrine cells. Although typically thought to have narrow processes (34), these cells may contribute to the distal activation of RGCs through connections with wide-field amacrine cells (17, 35). Wide-field amacrine cells, whose processes can span millimeters in the retina (36–39), may underlie the distal responses. Using strychnine (1 µM) to block glycine receptors while presenting a moving bar stimulus, we observed a significant decrease in the PRE response in ON RGCs (Fig. 6 *A*–*D*; 8.09 ± 0.85 spikes before and 3.63 ± 1.34 spikes after drug administration in the PRE preferred direction, respectively; *P <* 0.05, two-sided Wilcoxon signed-rank test; n = 15, mean ± SEM). Notably, this decrease did not result from an overall reduction in RGCs’ activity, as responses in the Central area slightly increased (Fig. 6*D* *Bottom*; *P* < 0.05, two-sided Wilcoxon signed-rank test). We also verified that the spike shape of recorded cells did not change before and after drug application (*P* < 0.001, shuffling analysis; *Materials and Methods*), confirming consistent recordings and ruling out erroneous spike sorting due to the drug.

Wide-field amacrine cells can contribute to the PRE response not only via glycinergic amacrine cells but also via their electrical coupling with RGCs (40–43). Blocking gap junctions using MFA (100 µM) significantly reduced PRE responses (*SI Appendix*, Fig. S7) and made ON RGCs responses more transient in the Central area. This suggests that gap junctions contribute both to the distal and classical RF responses in ON PRE RGCs (*Discussion*).

The model presented in Fig. 6*E* represents the simplest circuit based on the observed results: Narrow-field ON glycinergic amacrine cells provide an asymmetric tonic inhibition to ON PRE RGCs. Wide-field amacrine cells located in the activation zone inhibit these glycinergic cells directly or through bipolar cells (44–46), thereby exciting RGCs via disinhibition. In addition, wide-field amacrine cells may directly activate ON PRE RGCs through gap junctions. This circuitry is likely more complex, involving additional interneurons and other mechanisms enhancing response asymmetry (i.e., desensitization).

### PRE Responses Are Detected in the Visual Thalamus.

**Fig. 7.**
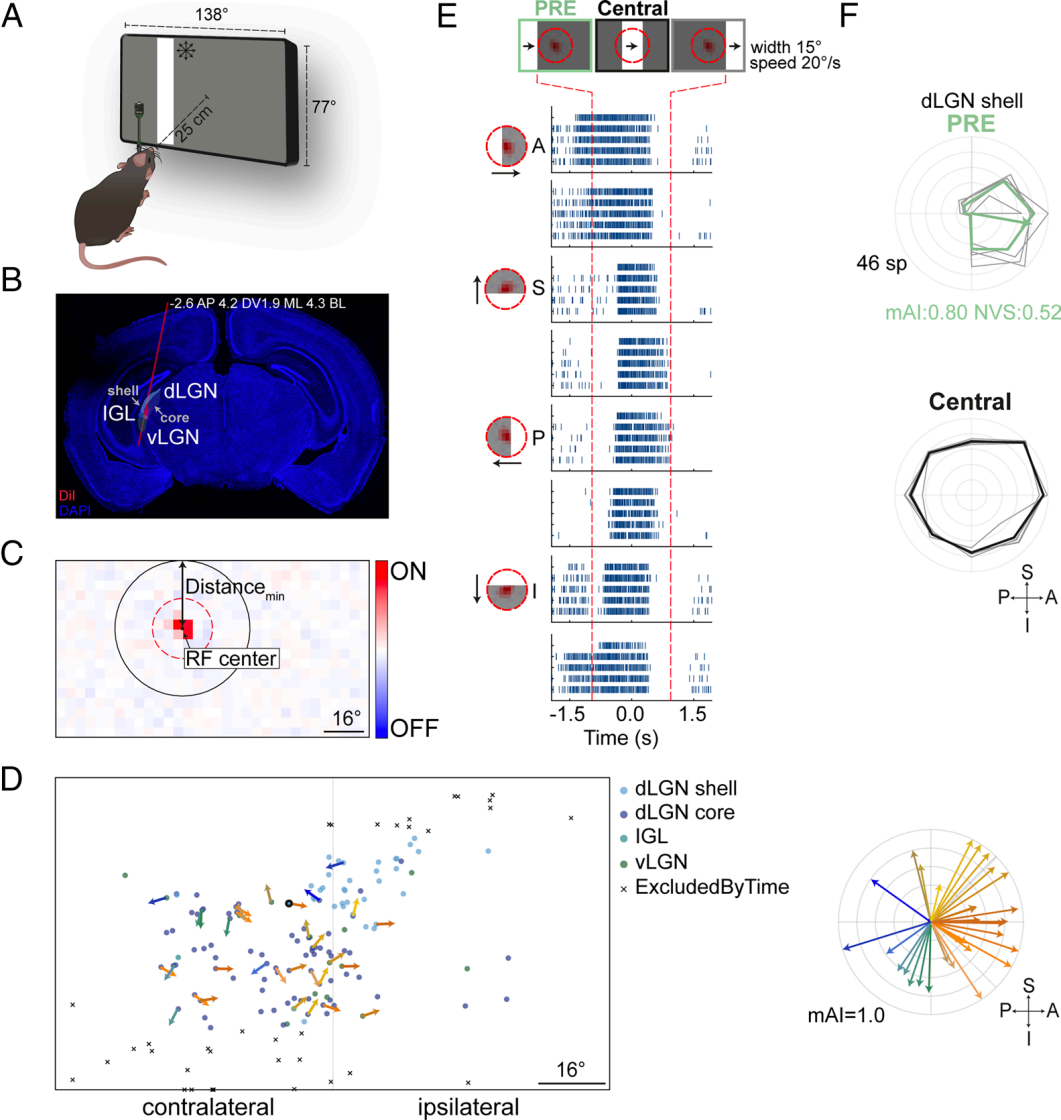
Extraclassical asymmetric responses in the mouse LGN. (*A*) In vivo recording setup schematic. (*B*) Coronal brain slice with probe track (red) overlaid. LGN areas are depicted. (*C*) RF of an example dLGN neuron in response to white-noise stimulation. The dashed, red circle (11.7° radius) around the cell delimits a fixed Central area. The minimum distance from the RF center to the closest screen border (Distance_min_) is depicted. (*D*) *Left*: RF center locations for recorded LGN neurons (n = 126), color-coded by anatomical region. Example neuron from *C* is circled. *Right*: Polar plot showing preferred directions for asymmetric PRE LGN neurons (19 and 13 out of the 55 and 71 included cells, respectively). The arrow length represents the mAI pointing toward the preferred direction. (*E*) Raster plots of the example cell from *C* in response to bars moving in 8 directions. Notations as in Fig. 1*D*. (*F*) Polar plots showing the dLGN neuron’s response to moving bars in the PRE (light green) and Central (black) areas. Notations as in Fig. 1*E*. Abbreviations: A, anterior; S, superior; P, posterior; I, inferior; dLGN and vLGN, dorsal and ventral LGN; IGL, intergeniculate leaflet. Directions are in world-centric coordinates.

The asymmetric PRE response observed in RGCs has been recorded ex vivo in the isolated retina. To determine whether these responses are being transferred to downstream retinal targets, we conducted in vivo experiments in anesthetized head-fixed mice using a Neuropixels probe and recorded from the LGN of the thalamus, a primary target of RGC projections (Fig. 7 *A* and *B*). LGN neurons’ RFs were identified using white-noise stimulation (Fig. 7*C*). To detect asymmetric PRE responses, we presented moving bars. Cells with RF centers located near the screen edge were excluded (*Materials and Methods* and Fig. 7*D* (left), cells depicted with a black cross), as this would prevent us from detecting light responses prior to the bar’s entry to their RFs. Consistent with our findings in RGCs, we identified LGN neurons exhibiting PRE asymmetric responses (Fig. 7 *E* and *F*) in both the dorsal and ventral LGN (dLGN and vLGN) and the intergeniculate leaflet (IGL) (Fig. 7*D*). Interestingly, while previous studies suggest that DSGCs mainly project to the LGN shell (47–49), our results demonstrate that information about direction of motion could be encoded by neurons in both the dLGN shell and core in their extraclassical RF.

## Discussion

We reveal that a subset of non-DS RGCs in the mouse retina utilize areas beyond their classical RF boundaries to encode motion direction. Our results indicate that the PRE response originates from an asymmetric distal area—the activation zone. The preferred directions of PRE responses are organized according to retinal location, revealing an overrepresentation toward the optic disc. These DS-like distal responses rely on two mechanisms. First, desensitization weakens the response when the bar moves in the PRE null direction. Second, the response to stimulation in the activation zone has an inherent DS component. The asymmetric PRE response is independent of speed, and pharmacological manipulations suggest that glycine and potentially electrical coupling contribute to the extraclassical response. Asymmetric PRE responses were also found in LGN neurons, suggesting transmission to downstream visual pathways.

### Mechanisms Underlying Extraclassical Asymmetric Surround Responses in RGCs.

The asymmetric PRE responses are distinct from classical surround responses. First, unlike the antagonistic center-surround RF organization, PRE responses in ON RGCs have the same ON polarity. Second, they emerge from light stimulation outside the classical RF of RGCs (>350 μm). PRE responses may be related to the periphery effect, where stimulation in the far periphery can modulate the activity of RGCs (10, 11, 13–15, 50–54). A plausible origin for the periphery effect is wide-field amacrine cells, whose processes can span a few millimeters of the retina (36–39, 42, 55). When displaying an asymmetric origin, such long-range inputs may underlie asymmetric responses.

Although most cells show increased PRE PD activity (Fig. 1*F* and *SI Appendix*, Fig. S2*E*), a minority (8.1%) exhibit baseline or lower activity. Thus, the circuitry underlying asymmetric responses in the extraclassical RF may involve a combination of asymmetric excitation and symmetric inhibition, in line with the previously described extraclassical suppression (13–15).

Our pharmacology experiments provide a step toward understanding the neuronal circuits connecting the distal wide-field amacrine cell and ON PRE RGCs. The results suggest a role for glycinergic amacrine cells and gap junction coupling in this circuit. Previous studies showed that glycinergic amacrine cells, themselves inhibited by wide-field amacrine cells, may activate RGCs in response to a far-away stimulation via disinhibition (17, 35). Electrical coupling between wide-field amacrine cells and RGCs was also reported before (40, 42). Particularly, it was shown that wide-field amacrine cells form gap junctions with M2 RGCs (56–58), and our examination of the RGC typology project suggested that M2 RGCs (25) belong to the ON PRE RGCs population. Nevertheless, we cannot exclude the possibility that gap junctions contribute to the asymmetric PRE response via other interneurons (41). In addition, MFA application, did not only eliminate PRE responses but also significantly reduced center RF activity. MFA is frequently used to investigate the physiological functions of electrical synapses and is considered among the more specific gap junction blockers (59, 60), but these results should be carefully interpreted, as the locus of MFA activity cannot be identified and off-target effects cannot be ruled out (61–63).

### Desensitization Mechanisms and an Inherent DS Component Contribute to Extraclassical Responses.

Desensitization contributes to make the response in the activation zone selective to the direction of motion. Desensitization mechanisms were previously described in the retina and other sensory systems and may involve desensitization of receptors, changes in the internal calcium concentration and other molecular processes (64–68). Using masking experiments (Fig. 4), we found an inherent DS component in the activation zone’s response that operates alongside desensitization to support encoding of motion direction in the extraclassical RF. The masking of the center RF allowed us to better understand the DS nature of the activation zone. In the unmasked experiments, we compared PRE PD and POST ND responses, but there is a limitation to this comparison – the POST response is calculated after the leading edge of the bar has already traversed a portion of the activation zone (*SI Appendix*, Fig. S6). Therefore, the time frame during which the POST response to the bar’s leading edge occurs was omitted from our analysis. This exclusion was necessary for the unmasked experiments because it coincided with the presence of the trailing edge within the classical RF, making it difficult to isolate the extraclassical response in the POST period from the classical RF response, especially with wider bars (Figs. 2&3). Masking experiments avoided this overlap, isolated the POST response and prevented desensitization, thereby enabling a balanced comparison. Even in this context, results showed stronger PRE than POST responses, confirming that the activation zone has an inherent DS component alongside desensitization. The finding that the response to stimulation in the activation zone is DS is in line with previous findings showing that distal inputs from wide-field amacrine cells may be directionally tuned (69, 70). Interestingly, it was shown that wide-field amacrine cells are also part of the classic DS circuit (54, 71–73).

Finally, although asymmetric PRE RGCs differ from classic DSGCs in their mechanisms and RF characteristics, they too encode motion direction in the visual field. Notably, even the PRE & POST RGCs subpopulation may carry information on motion direction when considering the entire trial – the full trajectory of the moving bar. When an object continuously moves across the visual field, a response prior to the object’s arrival in the center RF indicates motion in the PRE preferred direction. A response elicited after center RF stimulation indicates motion in the PRE null direction. Thus, the timing of the extraclassical response relative to the center RF response can provide information about motion direction.

### Extraclassical Responses Across the Visual System.

Not exclusive to the retina, periphery effects, or extraclassical RF, were reported in other stages of the visual pathway, including the LGN (74–77) and visual cortices (78–81). Already at the retina level, the extraclassical RF stimulation has complex effects. It can suppress or facilitate response to center RF stimulation depending on the context (10, 11, 13–15). For example, some RGCs respond to motion in the RF center only if the wider surround does not move with the same trajectory (50, 54, 82). Periphery stimulation can also mildly affect RGC activity in the absence of a central stimulus (83–89). The complexity of the extraclassical stimulation effect may emerge from its selectivity to stimulation parameters, such as orientation, spatial frequency, and speed (87, 90–95). The periphery effect along the visual system may be attributed to the retina (96), but additional central mechanisms were suggested to contribute (97). Our finding that LGN neurons exhibit similar asymmetric extraclassical responses indicates that it is transferred to downstream targets. This suggests that various neurons along the visual pathway, other than the classical DSGCs, may contribute to the encoding of motion direction.

### Comparison to Previous Studies.

The surround of RGCs is typically thought to be symmetric. Yet, prior studies have reported on asymmetric surrounds in retinal processing. First, it has been shown that RF structure may vary with retinal topography, revealing asymmetric surround at the visual horizon to enable efficient encoding of panoramic natural scenes (98). Additionally, asymmetric surround inhibition was shown to be critical for DS responses in noncanonical DSGCs (22, 99). Our findings extend this concept by demonstrating that the asymmetric surround not only contributes to DS computations but can operate specifically within the extraclassical RF.

Unlike prior studies that used linear or radial gratings over large visual field areas to elicit periphery effects, two recent studies employed moving bars to uncover extraclassical RF responses. One study demonstrated that salamander RGCs can encode motion from a distal bar (16), while another showed that in the rat retina, distal sensitivity is specific to fast OFF RGCs and emerges from changes in the bar’s speed (17). Both studies reported distal sensitivity without a directional component. In contrast, our findings in the mouse retina reveal that sustained ON RGCs exhibit sensitivity to distal motion that is both spatially restricted and DS. It is plausible that the speed sensitivity observed in rat retinas also involves a DS component.

### Potential Implications of the Asymmetric PRE Response.

While asymmetric PRE responses are generally weaker than Central responses, they can still convey meaningful information even at low firing rates. In the visual system, RGC firing rates often correlate with stimulus intensity, resulting in weaker responses to dim light (4). Other modalities also show this property. For example, in large environments, hippocampal place cells’ activity can vary by tenfold or more (100). In the auditory system, neurons encode low sound levels via phase-locked responses despite low firing rates (101). A major implication of the difference between asymmetric PRE and classical RF response magnitudes probably emerges during naturalistic stimuli, where classical and extraclassical RF are simultaneously stimulated. While a stimulus in the center RF may overrule responses in the extraclassical RF, a distal object can elicit an informative response on the motion direction in the absence of a preferred stimulus in the center RF.

The asymmetric PRE response, whereby stimuli far away from the classical RF activate RGCs, may complicate the known retinotopic organization (102). Nevertheless, our data demonstrate that these responses are more prominent during motion and are arranged in a centripetal pattern. With the optic disc near the optic axis (103), this spatial organization aids in detecting moving objects before they enter the classical RF, helping to prioritize critical visual information. By allowing early motion detection, this response may overcome the slowness of phototransduction and synaptic transmission processes, which delay the RGC response by up to 100 ms, improving real-time responses to rapidly moving stimuli and tracking their trajectories. An early anticipation was previously described in various species, where RGCs respond faster to moving than to flashing objects, enabling neurons to represent the actual position of the moving object accurately (31–33). This anticipation has no directional preference, although anticipatory mechanisms are also implemented by some DSGCs (104). Unlike these studies, the asymmetric PRE response we describe occurs before an object enters the RGC’s RF. This early anticipation may allow downstream structures to overcome additional accumulating delays along the visual pathway, enabling mice to better predict and respond to changes in their environment, which is crucial for survival.

## Materials and Methods

### Materials availability.

This study did not generate new unique reagents.

### Experimental Model, Method Details, and Analysis.

Experiments were performed on C57BL/6JOlaHsd wildtype mice. All experimental procedures were approved by the Institutional Animal Care and Use Committee (IACUC) at the Weizmann Institute of Science. Detailed materials and methods are described in Supporting Information.

### Ex vivo MEA Recordings.

Retinal preparation, MEA recordings, and spike sorting were performed as previously described (105–110). At the end of each experiment, a picture of the recorded retina laying on the electrodes was taken. Retinal borders and the outline of the recorded region were defined to determine the distance between the center of each cell’s RF and the retinal edges. Visual stimuli presented during the experiments consisted of (1) a white-noise checkerboard pattern; (2) a full-field flashed spot; (3) square-wave gratings; (4) a moving bar at varying speeds. A moving bar was also tested with parallel masks; (5) static bars. For pharmacology experiments, we used strychnine (1 µM, Sigma-Aldrich Cat# S0532) or MFA (100 µM, Sigma-Aldrich Cat# M4531).

To quantify extraclassical RF responses, we used a bar stimulus moving across the retina. RF centers were estimated using the white-noise stimulus, and the maximum circular area was defined by the minimum distance to retinal edges (Distance_min_). Only cells with a minimum Distance_min_ of 450 µm were included to ensure at least a 100 µm annulus for extraclassical RF quantification. The Central area was defined as a 350 µm radius around the RF center, with responses beyond this classified as extraclassical.

### In vivo Neuropixels Recordings.

In vivo recordings and spike sorting were performed as previously described (106). A Neuropixels 1.0 probe (111) was inserted to the LGN. Analysis included only units recorded in the dLGN, vLGN, and IGL. Visual stimuli included (1) a white-noise checkerboard and (2) a moving bar.

The PRE response analysis followed the same approach as for the MEA recordings, excluding cells near the screen border.

## Supplementary Material

Appendix 01 (PDF)

## Data Availability

MEA data and the code to produce the figures have been deposited in Zenodo https://doi.org/10.5281/zenodo.13119490 (112). Further information and requests for resources should be directed to and will be fulfilled by the lead contact, Michal Rivlin (michal.rivlin@weizmann.ac.il).
