# Supplementary material for: Retinal ganglion cells encode the direction of motion outside their classical receptive field
Source: Proc Natl Acad Sci U S A. 2024 Dec 30;122(1):e2415223122. doi: 10.1073/pnas.2415223122 (PMC11725840; doi:10.1073/pnas.2415223122)
Supplement: Supplementary file 1 — Appendix 01 (PDF) [file pnas.2415223122.sapp.pdf]

PNAS

Supporting Information for

Retinal ganglion cells encode the direction of motion outside their classical receptive field

Serena Riccitelli[a,1,2*], Hadar Yaakov[a], Alina S. Heukamp[a], Lea Ankri[a] and Michal Rivlin-Etzion[a,2,*]

[a] Department of Brain Sciences, Weizmann Institute of Science, Rehovot 7610001, Israel
[1] Present address: IRCCS Ospedale Policlinico San Martino, Genova, 16132, Italy
[2] To whom correspondence may be addressed. Email: serena.riccitelli@weizmann.ac.il (ORCID: 0000-0003-4369-4586) or michal.rivlin@weizmann.ac.il (ORCID: 0000-0002-1310-3370)

This PDF file includes:

Detailed Materials and Methods
Supporting Information text
Figures S1 to S7
SI References

Detailed Materials and Methods

EXPERIMENTAL MODEL

Experiments were performed on C57BL/6JOlaHsd wildtype mice (8-20 weeks old, both males and females). Weaned mice were kept on a 12h/12h light-dark cycle with food and water provided *ad libitum*. All experimental procedures were approved by the Institutional Animal Care and Use Committee (IACUC) at the Weizmann Institute of Science.

METHOD DETAILS

Retina preparation

Mice were kept under dark-adapted condition for 1 h, then anesthetized with isoflurane (Terrell, Piramal Critical Care Inc.) and decapitated. Eyes were immediately enucleated and dissected in a Petri dish containing AMES medium (Sigma, St. Louis, MO, USA) supplemented with 1.9 g/L sodium bicarbonate equilibrated with carboxygen (95% O_2 and 5% CO_2). Retinal tissue was isolated under a dissecting microscope. The orientation of the retina was determined based on landmarks on the choroid (1). Retinas were either dissected into two halves along the nasal-temporal axis (11 experiments) or kept whole (6 experiments). Because of asymmetric opsin distribution, with cones in the ventral retina being more UV sensitive (2), only dorsal retinas, except for an entire one, were recorded. Next, the tissue was placed on the multi-electrode array (MEA), pre-coated with a poly-D-lysine solution (PDL, 1.0 mg/ml in H_2O, Merck-Millipore, Cat# A-003-E) for 1h at RT, with the retinal ganglion cell (RGC) layer facing the electrodes (3). All procedures were performed in dim red and infrared light, and the room was dark throughout the experiment.

Ex vivo multielectrode array (MEA) recordings

MEA recordings were conducted on isolated retinas using MEAs of 252 electrodes (MultiChannel Systems, 30 µm diameter, 100 or 200 µm minimal electrode distance) while projecting light stimuli focused on the photoreceptor layer, as previously described (4–6). Briefly, the retina was constantly perfused using a peristaltic pump with an oxygenated bicarbonate-buffered AMES medium at a flow rate of 3.5 ml/min, and the temperature was maintained at 33.2°C. Data acquisition began 1 h after the retina was placed in the chamber to allow spike amplitude stabilization. Extracellular voltage signals were amplified, digitized at 20 kHz, and stored for offline analysis. Spike sorting was performed offline using Kilosort2.0 (7), followed by manual curation in Phy (8, 9). Only well-separated units with consistent spike shapes (72 datapoints) and refractory period violations <1% (10) were included. After each experiment, a picture of the recorded retina laying on the electrodes was taken, and retinal borders and the outline of the recorded region were defined to determine the distance between each cells' RF center and the retinal edges. RF centers from white-noise stimulation in OLED coordinates (see *Analysis of MEA recordings*) were mapped onto retinal image coordinates. The retinal edges overlaid on the MEA are shown for some example cells (Figs. 1B, 3B, S1 and S2). The optic disc position and

nasal-temporal cuts were used to align flat-mounted retinas and map RGCs location to the retinal coordinate system (Figs. 1H-J).

Visual stimuli used in MEA experiments

Visual stimuli were created in MATLAB (R2018a) using Psychophysics Toolbox (Brainard, 1997; Pelli, 1997) and projected onto photoreceptors via a monochromatic OLED display (eMagin, EMA-100309-01 SVGA+, 600x800 square pixels, 60 Hz refresh rate) through a telecentric lens (Edmund Optics, 2.0X, Cat# 58-431). Pixel size on the retina was 7.5 μm, with irradiance ranging from 7.04 $R^{*}rod^{-1}s^{-1}$ to $2.43x10^3$ rod isomerizations ($R^{*}rod^{-1}s^{-1}$). Stimuli included: (1) a 15-minute checkerboard white-noise stimulus consisting of black-and-white squares (100% contrast), 60 μm^2 in size, changing at 30 Hz; (2) a flashed spot stimulus consisting of 3 s black, 2 s white, and 3 s black was presented over the entire retina (full-field, radius 1125 μm); (3) square-wave gratings (in all except one experiment) of 100% contrast and a spatial frequency of 397.5 μm (13.25°) that moved at a speed of 795 μm/s (2 Hz, 26.5°/s), presented in 8 pseudo-random directions; (4) a moving bar stimulus consisting of a white bar (300 or 900 μm width x 2500-3750 μm length, corresponding to 10° or 30° width x 83-125°) on a black background that moved at a speed of 600 μm/s (20°/s) with a 2025-2700 μm (67.5-90°) circular radius mask, presented in 8 pseudo-random chosen directions, in 45° intervals. The long axis of the bar was oriented parallel to the direction of movement. The moving bar appeared outside the retina and slid across it to ensure that neuronal activity was in response to the motion rather than a flash response. Each trial was preceded by a 500 ms period of the stimulus background, which was used to assess RGCs' baseline activity and followed by an additional 500 ms to allow the cell to return to a baseline state. The same moving bar stimulus (white bar, 900 μm width x 3750 μm length) was repeated in 5 experiments with masks (at background light level; 700 μm width x 2500 μm length) oriented parallel to the moving bar axis (see Fig. 4B). Note that the mask's width is comparable to the Central area size and each mask orientation was presented in 9 or 13 different positions spaced 200 μm or 300 μm (center-center) in a pseudorandom order. Same mask locations were chosen for opposite directions. In 6 out of 17 experiments, the bar stimulus (900 μm) was presented at different speeds (400, 600, 800, and 1000 μm/s). (5) Static 300-μm-wide white bars on a black background (250 ms black, 500 ms white, 250 ms black) were presented at locations spaced 75 μm apart across four axes (the same used for the motion stimuli). Both the direction and the offset were pseudo-random. Stimuli (except for the white noise) were repeated at least 4 times.

Pharmacology in MEA experiments

Responses to visual stimuli were recorded both without drugs and either with strychnine (1 μM, Sigma-Aldrich Cat# S0532) or MFA (100 μM, Sigma-Aldrich Cat# M4531) in 4 and 3 experiments, respectively. Strychnine/MFA was washed in for at least 15/20 min before repeating the visual stimuli again. Solutions were prepared fresh for each experiment.

In vivo Neuropixels recordings

In vivo recordings and spike sorting were performed as previously described (5). At least three days before recording, mice underwent acute surgery to implant head bars for head fixation during electrophysiological recordings. Under isoflurane anesthesia (5% induction and 1.5-2.5% maintenance, SomnoSuite (Kent Scientific)), the skin was cut away, and a metal frame was glued to the skull and a craniotomy was prepared to access the LGN. Mice were sedated with an intraperitoneal injection of Chlorprothixene (Sigma Aldrich), then anesthetized with Urethane (intraperitoneal, Sigma Aldrich) and maintained at 37°C on a feedback-controlled heating pad. After securing the mouse on a stereotactic device (Scientifica), the scalp was removed and the brain exposed before inserting a Neuropixels probe ("Neuropixels 1.0") (11) through 1-2 mm diameter craniotomy, drilled 2.5-2.7 mm posterior to bregma and 1.9-2.2 mm lateral, to a depth of 3.7 mm. After reaching the desired depth, the probe was allowed to settle before starting the recording session, which lasted ~2 h. The probe's tip was dipped into a 1 µL droplet of CM-DiI to facilitate probe tracking during imaging (see *Tissue processing and microscopy section*). A thin layer of paraffin-based transparent ophthalmic ointment was applied (Duratears, Alcon) to prevent the eyes from drying.

A black curtain was lowered over the rig, keeping the mice in complete darkness except for the visual stimulus. Neuropixels data were acquired at 30 kHz (spike band) using SpikeGLX (https://billkarsh.github.io/SpikeGLX) and processed with Kilosort2 to identify spike times and assign spikes to individual units (7, 12), with subsequent manual curation in Phy (8, 9).

Pupil movement was monitored in head-fixed, anesthetized mice, confirming that the visual stimuli did not activate the optokinetic reflex or induce pupil movement, consistent with (13).

Visual stimuli used in Neuropixels experiments

Visual stimuli were generated using MATLAB custom scripts based on Psychophysics Toolbox (Brainard 1997, Pelli 1997), displayed using an LG LCD gamma-corrected monitor (1280 x 720 pixels, 60 Hz refresh rate), positioned 25 cm from the mouse and spanning 137.6° azimuth x 77.4° elevation of its visual field. Stimuli were presented binocularly. With 1 degree in the visual field covering 30 µm on the mouse retina, the pixel-to-degree ratio was approximately 9.3. Experiments began with a receptive field mapping stimulus consisting of a 15-minute checkerboard white-noise stimulus (4.3° square size, 15 Hz). Next, mice were shown a moving bar stimulus consisting of a white bar (15° width x 215° length) on a black background, moving through the center of the screen in 8 pseudo-random directions, in 45° intervals, at 15°/s with an 86° circular mask (corresponding to 450 µm bar width moving at 600 µm/s on the retina). Each stimulus was repeated 5 times.

Tissue processing and microscopy

After recording, probes were retracted and mice were deeply anesthetized with a terminal intraperitoneal injection of pentobarbital (Pentobarbital Sodium, 200 mg/ml, CTS Chemical Industries Ltd., Kiryat Malachi, Israel), and perfused with phosphate-buffered saline (PBS, Biological Industries Israel, Cat# 02-023-1A, pH 7.4) and 4% paraformaldehyde (PFA, ChemCruz, Santa Cruz Biotechnology, Inc., Cat# 30525-89-4). Extracted brains were fixed further in 4% PFA for 24-48 h, washed in PBS, and sliced (30 µm) using a vibratome (7000 smz-2 Vibratome, Campden Instruments

Ltd.). Slices were mounted onto Superfrost/Plus Microscope Slides (Thermo Scientific), covered with a coverslip using a Vectashield antifade mounting medium with DAPI (Vector laboratories, H1200). All brain sections were digitally scanned using Olympus UPlanSApo 10x/0.40 NA objectives on an Olympus BX61VS slide scanner (Olympus Corporation, Tokyo, Japan). Further image processing was performed with Fiji software (14). Reconstruction of the fluorescent probe track was obtained in coronal slices using the SHARP-Track tool (https://github.com/cortex-lab/allenCCF) from (15) and each point along the probe was translated into the Allen Institute Common Coordinate Framework (CCFv3) template brain. Each CCFv3 coordinate corresponds to a unique brain region, identified by its structure acronym (e.g., CA3, TH, fp, *etc.*). Only units recorded in the dLGN, vLGN, and IGL were used for further analysis.

QUANTIFICATION AND STATISTICAL ANALYSIS

Analysis of MEA recordings

Full-field spot

To assess RGC polarity preference, an ON-OFF index (OOI) was calculated from responses to a full-field spot stimulus: $OOI = \frac{R_{ON} - R_{OFF}}{R_{ON} + R_{OFF}}$, where R_{ON} and R_{OFF} represent the spike count during 2 s of light ON or OFF, respectively. OOI values range from -1 to 1, with RGCs classified as ON (OOI≥-0.3), OFF (OOI≤-0.3), and ON-OFF (-0.3<OOI<0.3). The peristimulus-time-histogram (PSTH) was averaged across repetitions using a bin width of 10 ms. RGCs were identified as transient or sustained based on response duration, calculated from the number of PSTH bins exceeding the mean baseline firing rate±3*standard deviations (SDs) following light onset or offset for ON and OFF responses, respectively. Baseline firing rate was calculated over 60 s before the stimulus. Responses lasting longer than 500 ms were classified as sustained. ON RGCs were grouped into 5 clusters via principal component analysis (PCA) followed by k-means clustering of z-scored mean PSTHs calculated in response to the full-field spot. Standard response metrics, including baseline activity, peak response, latency to peak, OOI, response duration and RF diameter (see *White-noise*) were compared across clusters (Fig. S5H). A second dataset of ON PRE RGCs recorded while masking the Central area (Fig. 4F) were sorted into the existing clusters from Fig. S5G (Fig. S5I). This classification was based on the Pearson correlation between each cell response to full-field spot and the mean cluster responses, assigning cells to the cluster with the highest correlation (4).

White-noise

RGC RF centers were identified from the spike-triggered average (STA) from the white-noise data, averaging frames within 500 ms (in time steps of 20 ms) before each spike. The frame with the highest peak-to-peak amplitude was used to fit a 2D Gaussian, defining RF diameter as 2 SDs. The temporal component (Fig. S5C) was derived by averaging pixel values within the RF center. RGCs were included if they had a firing rate ≥ 1 Hz, RF diameter <500 µm and a clear negative or positive peak within 250

ms from a spike. For cells not meeting these criteria (22 out of 272 PRE RGCs), RF centers were assigned to the recording electrode position (see below).

Moving gratings and bars

Direction-selective ganglion cells (DSGCs) were identified using response to moving gratings. A normalized vector sum (gDSI) was calculated as: $gDSI = \frac{|\Sigma R_\theta e^{i\theta}|}{\Sigma R_\theta}$, where R_θ is the mean spike count for direction θ during the stimulus presentation. The preferred direction (PD) corresponded to the angle of the vector sum. The direction-selectivity index (DSI) was calculated as: $DSI = \frac{R_{PD} - R_{ND}}{R_{PD} + R_{ND}}$, where R_{PD} and R_{ND} are responses in the direction closest to the PD and its opposite, respectively. DSGCs were defined by gDSI≥0.15, DSI≥0.3 and mean firing rate >1 Hz. Moving bar stimuli produced broader tuning, classifying 5.0% of RGCs as DSGCs compared to 8.6% with gratings.

Definition and characterization of extraclassical RF responses using moving bar stimuli

To quantify RGC responses in the extraclassical RF, we used a bar appearing outside the retina and moving across it (Fig. S2A). The MEA technique allowed simultaneous recordings from spatially distributed neurons (Fig. S1A_i-D_i), so the position of the bar relative to each cell's RF was determined. RF centers were estimated and the maximum circular area was defined by the minimum distance to retinal edges ($Distance_{min}$ in Figs. S1A_i-D_i, 2A, C). The distances were calculated by determining the intersection points between the retinal edges and the path of the bar (leading edge) in each direction (Fig. S2A). For each cell, spike counts were only considered while the bar moved within retinal edges, ensuring that the time window was the same across directions. The Central area was defined as the region within a 350 µm radius from the RF center, with responses beyond this region classified as extraclassical. Only cells with a minimum $Distance_{min}$ of 450 µm were included, so the extraclassical RFs could be quantified over an annulus of at least 100 µm. The Central area radius was conservatively chosen, as it was well beyond the average RF size of RGCs (estimated at 139.4 ± 1.9 µm in diameter, mean ± SEM) and exceeds the largest RGC dendritic field radii (16–21). The inclusion criteria resulted in 90.4±2.7% of the total recorded cells for the 350 µm Central area radius (n=2207; mean±SEM). For the analysis described in Fig. S2D, we also considered Central areas of 250, 500 and 700 µm radii that resulted in the inclusion of, on average, 95.6±1.7%, 77.3±4.3% and 52.6±5.2% of the total recorded cells, respectively (mean±SEM).

The bar stimulus was aligned to each cell's RF center, so the time when the bar center is aligned with the RF center is defined as 0 (t_0 in Fig. S2A). Accordingly, the time when the bar's leading/trailing edge entered/left the extraclassical annulus ($-t_{Ex}$/$+t_{Ex}$, respectively; Fig. S2A) were considered for the analysis. The time when the leading/trailing edge of the bar entered/left the Central area was defined as $-t_C$/$+t_C$, respectively. PRE and POST responses included spikes occurring between {$-t_{Ex}$, $-t_C$} and {$+t_C$, $+t_{Ex}$}, respectively, except for Fig. 4F-D (see below and Fig. S6). PRE and POST responses were considered asymmetric if NVS≥0.15 (calculated as described above for the gDSI, with shuffle permutation test, 1000 permutations, α=0.05), motion asymmetry index, mAI≥0.3 (calculated as described above for the DSI) and minimum spike thresholds (≥3 spikes in half of the trials or 2 spikes

in all trials in 2 directions). Given that cells occupy different positions relative to retinal edges, PRE and POST responses were calculated using different radii for each RGC. Analysis was repeated with fixed annulus radii (350-700 µm or 500-700 µm) to verify consistency across RGCs. Similarly, we applied the criterion of including only cells where the annulus was within the retinal edges, resulting in an average of 66.5±4.7% (mean±SEM) of the total recorded cells.

The correlation between the mean spike shapes of PRE and Central responses was assessed by calculating the mean correlation coefficient for corresponding pairs. To assess statistical significance, a shuffling analysis was performed in which the Central response assignments were randomly permuted 1000 times. For each shuffle, the mean correlation coefficient was recalculated. The p-value was defined as the proportion of shuffled correlations that were greater than or equal to the observed mean correlation. Additionally, to verify that pharmacological manipulations (strychnine and MFA) did not affect spike shape, we performed a similar analysis comparing spikes shape waveforms before and after drug application.

To exclude light aberration as a cause for extraclassical RF responses, we conducted two sequential recording sessions. In the first session, we recorded responses to a set of stimuli, including a moving bar, and identified PRE RGCs. Then, we rotated the MEA by 180° and repeated the recordings to test whether the PRE PD of extraclassical RF responses remained consistent. We focused on cells recorded from the same electrodes in both sessions, allowing for a direct comparison before and after rotation (Fig. S4A).

The PRE- to-Baseline Response Index (PBRI) quantified activity changes for both PRE preferred and null directions relative to baseline as: $PBRI = \frac{R_{PRE} - R_{baseline}}{R_{PRE} + R_{baseline}}$. The resulting index provided a normalized measure that ranged from -1 to 1. A positive PBRI indicated increased activity during the PRE response.

Static vs. Moving bar analysis for extraclassical RF responses

For static bars, RGC responses to bars presented orthogonally to the preferred-null axis of the PRE response (determined from the moving bar stimulus, i.e., the orthogonal orientation of the static bar closest to PRE PD) were analyzed. Only bar positions outside the Central area and within the retinal borders were considered in calculating PRE and POST responses. The static asymmetry index (sAI) was calculated as: $sAI = \frac{R_{PS} - R_{NS}}{R_{PS} + R_{NS}}$, where R_{PS} and R_{NS} are the mean firing rates for bars on the preferred and null sides, respectively. Note that R_{PS} represents responses from various positions on the preferred side, which may involve partial coverage of the extraclassical RF, yielding to a weaker mean response. For moving bars, the PRE and POST PD responses were used to calculate the moving asymmetry index (mAI). sAI values range from -1 to 1. A value of 1 indicates the presence of an activation zone on the preferred side, with no response on the null side; 0 indicates symmetric activity, signifying the absence of an activation zone; and a negative value indicates stronger activity on the null side. To study RGCs' desensitization in the activation zone, trials were grouped based on the previous static bar location: either following Central area stimulation (all static bar orientations) or after random positions outside the Central area and the activation zone. The response in spikes/s on the preferred side was

calculated by averaging responses from the 'After Central area stimulation' and 'No previous stimulation' trials.

Comparison of masked vs. unmasked moving bar responses in extraclassical RF

To compare masked and unmasked moving bar responses in the extraclassical RF, the mask's position relative to each cell's RF center was determined by its distance to the mask's longitudinal axis passing through the center. In some cases, this resulted in an almost perfect match (the mask fully covers the Central area), while in other cases, the mask was slightly shifted relative to the RF center. Yet, as we used multiple masks presented at a maximum of 300 μm distance (center to center), the offset between the RF center and the central mask axis was <150 μm. The 700 μm wide mask covered at least a 200 μm radius from the RF center, fully covering the dendritic field of nearly all RGCs (16–21). Importantly, the mask was placed symmetrically for opposite directions, ensuring valid comparisons between the PRE PD and POST ND. PRE extraclassical responses were quantified as described and compared across conditions. Masking the Central area allowed for more accurate POST response quantification by capturing the bar's leading edge response, whereas unmasked trials only included responses after the trailing edge exited the Central area. PRE and POST responses in the masked condition were measured as the bar's leading edge entered and left the extraclassical annulus (Fig. S6).

Analysis of Neuropixels recordings

RFs of LGN cells were calculated based on STA from the white-noise data, similar to RGC RFs. Only cells with a minimum firing rate of 0.8 Hz, RF diameter <750 μm and a clear peak within 350 ms of a spike were included. The cell was excluded from the analysis when the RF assessment did not pass the quality check.

We used moving bars to quantify responses outside the Central area, excluding cells near the screen border (within 15°, corresponding to 450 μm on the retina, comparably to the *ex vivo* experiments), ensuring PRE responses were recorded over a minimal distance of 3.3°. The PRE response analysis followed the same approach as for the MEA recordings.

Statistics

Unless indicated otherwise, statistical parameters are reported in the text and figure legends, including the exact value of n (number of experiments and cells) and the mean ± standard error of the mean (SEM). Population data in Figs. 1F, G; 2F-H; 3G; 4A, F; 6D are shown as mean ± standard deviation (SD).

To compare differences in paired conditions, the Wilcoxon signed-rank test was used. The Friedman's and the Kruskal-Wallis test with Tukey-Kramer/Bonferroni correction were used to compare differences across groups. To compare differences in fractions, a Chi-square test was used. No statistical tests were performed to predetermine sample size. All statistical tests were two-sided. The CircStat toolbox (113) for MATLAB was used for the descriptive and statistical analysis of directional data.

Statistical significance was accepted at $p<0.05$. Notation of p-values follows: *$p<0.05$, **$p<0.01$, ***$p<0.001$. The statistical tests were performed using MATLAB 2018b and 2019b (Natick, Massachusetts: The MathWorks Inc).

Supporting Information text

Validation of PRE responses

We conducted a series of control analyses and experiments to confirm that extraclassical RF activity was not due to technical issues.

Although unlikely, light projected to one position of the specimen may be reflected to a different location (due to an air bubble or other distortion), which could lead to misinterpretation of the spatial origin that drives an RGC response. We excluded this possibility by two means. First, if the phenomenon resulted from light aberration, we would expect nearby RGCs to respond similarly to the same stimulus outside their classical RFs. To test this, we examined neuron pairs recorded on the same electrode. Of 169 RGCs showing a PRE response and having at least another RGC recorded on the same electrode (Fig. S3A), only 31 pairs showed asymmetric PRE responses (ΔPD=0.35±21.6°, mean vector ± standard angular deviation), suggesting that the PRE response is RGC specific rather than due to light aberration (Fig. S3B). Second, we examined the stability of PRE responses by rotating the retinal specimen while presenting the same stimulus. If light aberration underlined the PRE response, different retinal areas would be stimulated before and after the rotation, causing the PRE response to disappear or reverse after MEA rotation. Instead, ~55% of RGCs maintained their asymmetric PRE responses and directional tuning (significantly correlated, ρ=0.48 with $p<0.01$; ΔPD=-33.7±59.8°, mean vector ± standard angular deviation, n=45. There is not sufficient evidence to reject the hypothesis that the true population mean is equal to 0°, one-sample test for the mean angle) (Fig. S4, see Materials and Methods). We also excluded spike sorting errors, i.e., an erroneous grouping of spikes originating from two distal RGCs. This is unlikely, as two RGCs recorded on the same electrode typically exhibit overlapping RFs, although an electrode can also record the activity from an axon originating from a distal location. Comparing waveforms from the Central area and PRE responses, we found highly similar spike shapes, indicating they originated from the same unit ($p<0.001$, based on the mean correlation between corresponding pairs of the PRE and Central mean spike shapes compared to the distribution of shuffled mean correlations (see Materials and Methods); example mean spike shape for the PRE and Central spikes in Fig. S1A_{vii}, A_{viii}). Moreover, only cells with <1% refractory period violations, calculated across the entire experiment, were included (10). Together, we conclude that PRE responses reflect genuine responses to stimuli outside the classical RF of RGCs.

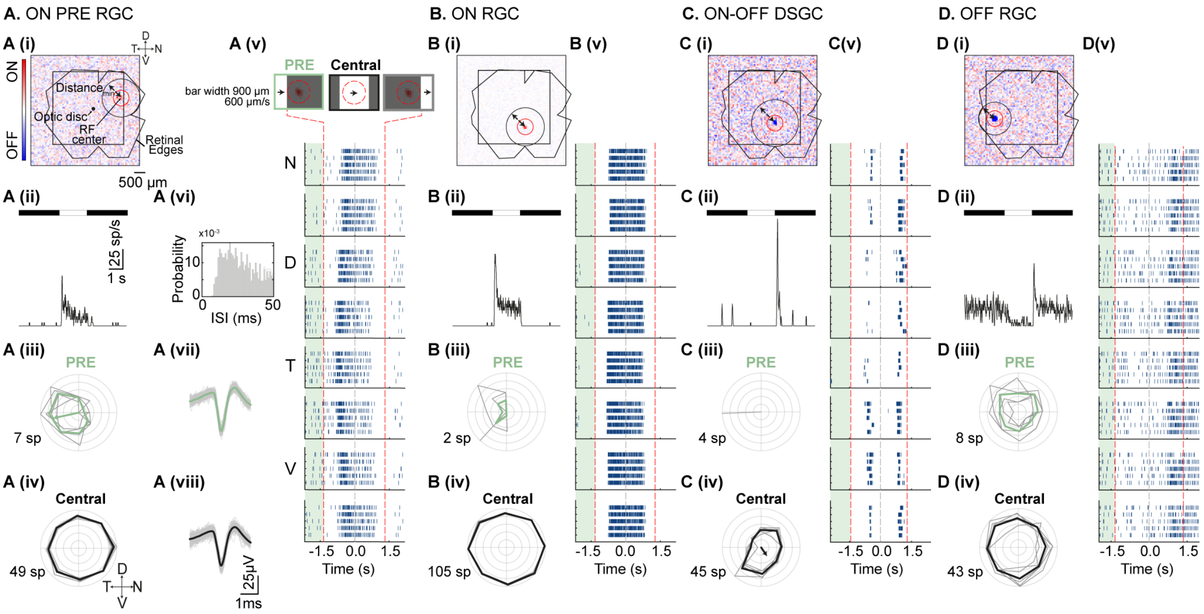

Fig. S1. RGCs' responses to a moving bar stimulus, related to Fig. 1. A. (i) For each RGC, Distance_{min} depicted by the black arrow, is defined as the maximal radius around the RF center that is included within retinal edges (distance from RF center to the closest retinal edge). We only included in our analysis RGCs with Distance_{min} > 450 µm. The red line depicts the Central area (350 µm radius). **(ii)** Peristimulus time histogram (PSTH, 5 repetitions, 10 ms bin) of the RGC in (i) showing its response to a full-field spot stimulus (indicated above). **(iii&iv)** Polar plots of the RGC in (i) in response to the moving bar, calculated before (PRE, light green) and while the bar crossed the Central Area (black). Bold lines show the mean response and thin lines represent single repetitions. The arrow points to the preferred direction, and its length represents the motion asymmetry index, mAI (iii), and direction selectivity index, DSI (iv) (outermost radius equals 1). **(v)** Raster plots of the example RGC in (i) in response to a bar moving in 8 directions (4 denoted on the left). Each line is a trial. An illustration of the bar's location relative to the RGC's RF center is illustrated above for different time points. The example shows that PRE responses emerged before the bar reached the Central area. **(vi)** The inter-spike-interval (ISI) calculated across the entire experiment for the RGC in (i). **(vii&viii)** Spike waveforms evoked in response to the light stimulation in the PRE (vii) and Central area (viii), respectively. Bold lines represent the mean, and gray lines represent a random subset of 250 spikes. **B, C, D. (i-v)** Same as in $A_{i\text{-}v}$ for three RGCs recorded within the same retina: ON RGC (B), ON-OFF DSGC (C), OFF RGC (D). The examples show that responses were elicited while the bar crossed the RF, with ON RGCs responding to the leading edge of the white bar, OFF RGCs responding to its trailing edge, and ON-OFF direction-selective RGCs (DSGCs) responding preferentially to the bar moving in their preferred direction. Retinal coordinates and scale as in A.

Abbreviations: D, dorsal, N, nasal, V, ventral, T, temporal, indicating the retinal coordinates; sp, spikes.

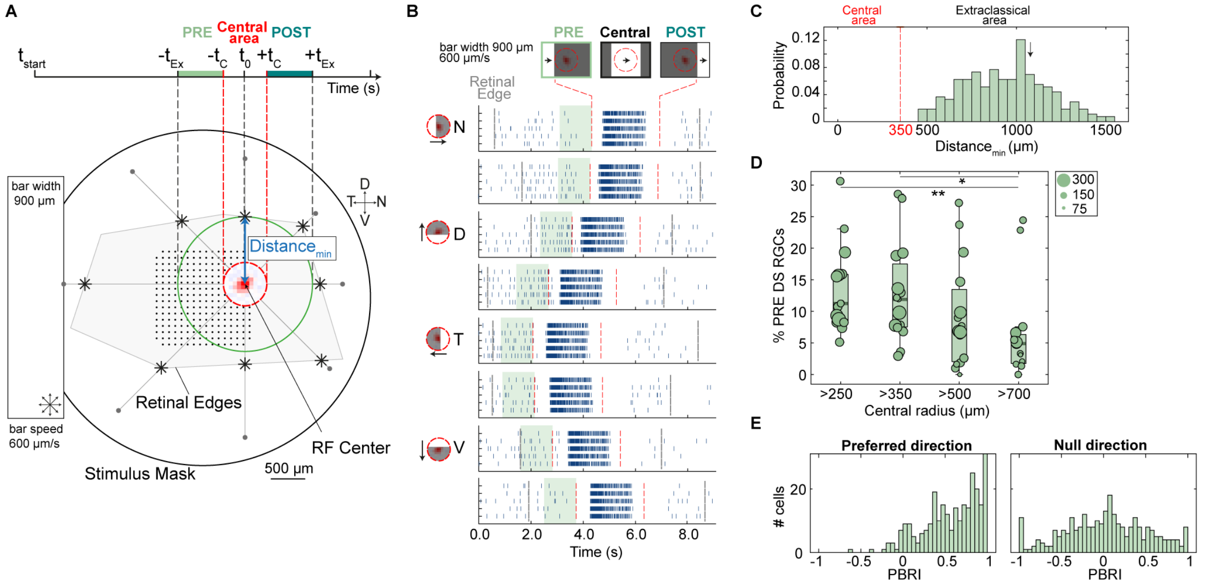

Fig. S2. Alignment of RGCs' responses to a moving bar stimulus: classical and extraclassical RFs definition, related to Fig. 1. A. Schematic of the moving bar stimulus. The stimulus mask determines the starting point of the bar outside the retina (to avoid a flash response) at time t_{start} and its entry into the Central area depends on the cell's location in the retinal specimen. Bar starting positions are depicted by gray dots and their trajectories with a gray line for 8 directions. The image illustrates the RF of the example PRE RGC from Fig. 1 computed in response to the white-noise stimulation. t_0, the time when the center of the bar is aligned to the RF center. The dashed, red circle (radius 350 µm) delimits the fixed Central area; $-t_C$ and $+t_C$ define the times when the bar's leading/trailing edge reaches/leaves the Central area, respectively. $-t_{Ex}$ and $+t_{Ex}$ indicate when the bar's leading/trailing edge reaches/leaves the extraclassical RF annulus. The extraclassical RF is defined according to the cell location as the maximal circular area around the RF center included within retinal edges (blue line, $Distance_{min}$). We only included in our analysis RGCs with $Distance_{min}$ > 450 µm. The intersection points between the trajectories of the bars (leading edges) and retinal edges are depicted by asterisks. Black dots represent MEA electrodes. **B.** Raster plots in response to a bar moving in 8 different directions (4 denoted on the left) of the example RGC in A before aligning the spiking activity to the RGC's RF center and trimming spikes that occurred when the stimulus was moving outside retinal edges. Each line is a trial. An illustration of the bar's location relative to the RGC's RF center is presented on top for the nasal direction for different time points. The time the leading edge of the bar stimulus reaches retinal edges, or the Central area is depicted in grey and red (dashed lines), respectively. Shaded light green delineates the time window used to calculate the PRE responses, and its size is determined by $Distance_{min}$. **C.** The distribution of the $Distance_{min}$ of all PRE RGCs (272 cells, 15 retinas). The gray arrow depicts $Distance_{min}$ of the example RGC. **D.** Percentages of RGCs presenting an asymmetric PRE response as a function of Central area size. Each dot represents a retina, and its size correlates with the number of included cells. The percentage of PRE RGCs is significantly smaller for distances >700 µm compared to the >250 and >350 µm radii (6.5±2.0% *vs*. 13.3±1.8 and 12.7±2.0%, respectively, mean±SEM). The percentages remained similar when we changed our inclusion criteria to a fixed extraclassical area for all cells (10.5±1.9% and 6.3±1.8% for an annulus between 350-700 µm and 500-700 µm around the RF center, respectively; see Materials and Methods). **E.** PBRI (PRE-to-Baseline Response Index) calculated in response to the moving bar in the PRE preferred direction (left) and the null direction (right).

Abbreviations: D, dorsal, N, nasal, V, ventral, T, temporal, indicating the retinal coordinates. (D) *, **: p<0.05, 0.01, respectively, according to the Kruskal-Wallis test with Tukey-Kramer correction.

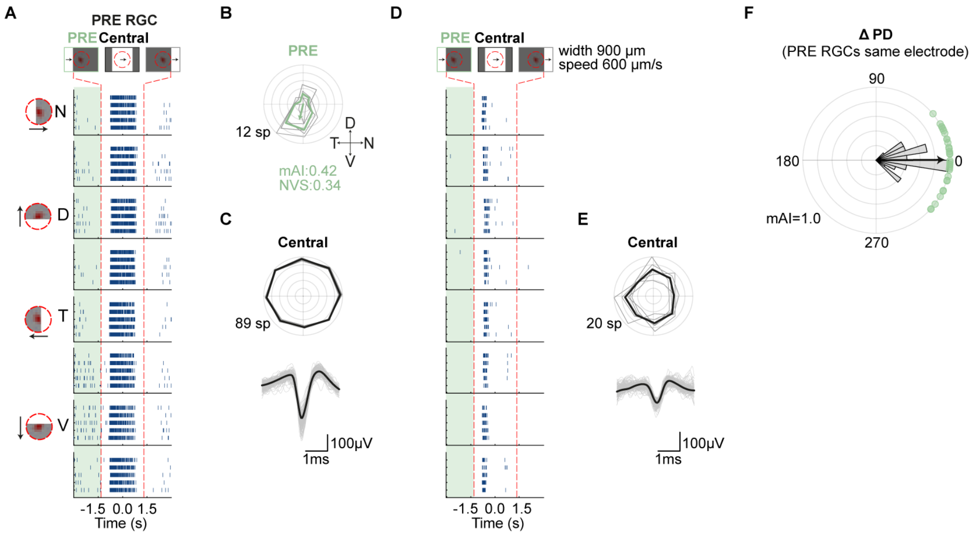

Fig. S3. RGCs presenting or not an asymmetric PRE response recorded on the same electrode, related to Fig. 1. A. Raster plots of an example PRE RGC in response to a bar moving in 8 directions (4 denoted on the left in A). Each line is a trial. The red lines confine the time when the bar crosses the Central area. An illustration of the bar's location relative to the RGC's RF center is presented on top. **B.** Polar plot of the PRE RGC (from A) calculated before the bar entrance into the Central area (PRE, light green). The bold line shows the mean response, and the thin lines represent single repetitions. The arrow points to the preferred direction, and its length represents the mAI. **C.** Top: As in B in response to the bar moving within the Central area (black). Retinal coordinates as in B. Bottom: Waveforms of the spikes evoked in response to the bar stimulus. Bold lines represent the mean, and gray lines represent a random subset of 250 spikes. **D, E.** Same as A, C for an RGC not presenting a PRE response recorded on the same electrode. **F** Polar plot showing the ΔPD for all pairs of PRE RGCs recorded on the same electrode (31 pairs, green circles). The circular histogram is overlaid. The arrow represents the mean vector (outermost radius equals 1).

Abbreviations: D, dorsal, N, nasal, V, ventral, T, temporal, indicating retinal coordinates; sp, spikes; mAI, motion asymmetry index, NVS, normalized vector sum.

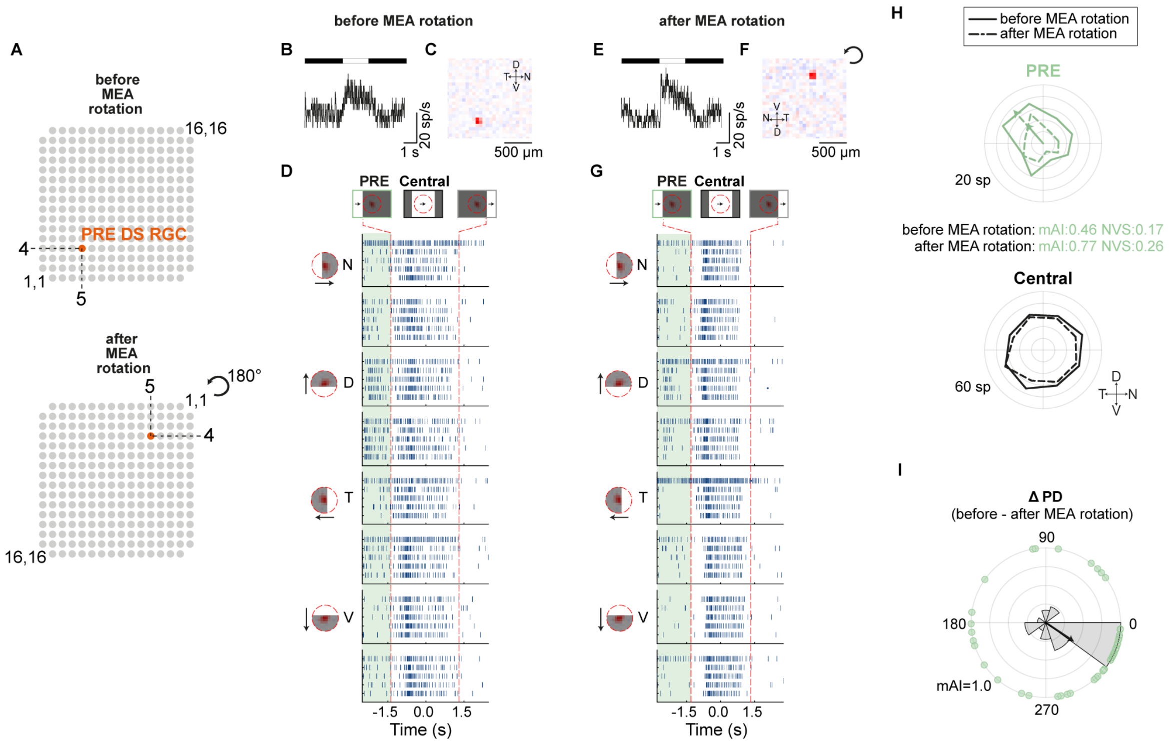

Fig. S4. Asymmetric PRE responses are maintained after MEA rotation, related to Fig. 1. A. Top: Schematic representation of the first session, where responses to a moving bar were recorded to identify PRE RGCs. Bottom: Illustration of the second recording session, showing the 180° rotation of the MEA. Responses from the same electrode were considered to test the consistency of PRE PD responses. Gray dots mark MEA electrodes, one example electrode is highlighted in red. **B.** Peristimulus time histogram (PSTH, 5 repetitions, 10 ms bin) showing ON response of an example RGC to a full-field spot stimulus (indicated above). **C.** RF spatial component of the example RGC in B computed in response to white-noise stimulation. **D.** Raster plot of the same RGC responding to a bar moving in 8 directions (4 are denoted on the left). Each line is a trial. The red lines confine the time when the bar crosses the Central area. An illustration of the bar's location relative to the RGC's RF center is illustrated above. **E-G.** Data from B-D repeated after rotating the MEA. Due to the similarity between the two example RGCs (before and after rotation), recorded from the same electrode, we suggest this is the same RGC. **H.** Polar plot of the PRE (top) and Central area (bottom) responses to the moving bar. The mean responses before and after MEA rotation are shown in continuous and dashed lines. Arrows point to the preferred directions, and their length represents the mAI (outermost radius equals 1). **I.** Polar plot of ΔPD for all PRE RGCs pairs recorded before and after MEA rotation (45 pairs, 3 experiments, green circles). The circular histogram is overlaid. The arrow represents the mean vector (outermost radius equals 1).

Abbreviations: D, dorsal, N, nasal, V, ventral, T, temporal, indicating retinal coordinates; sp, spikes; mAI, motion asymmetry index, NVS, normalized vector sum.

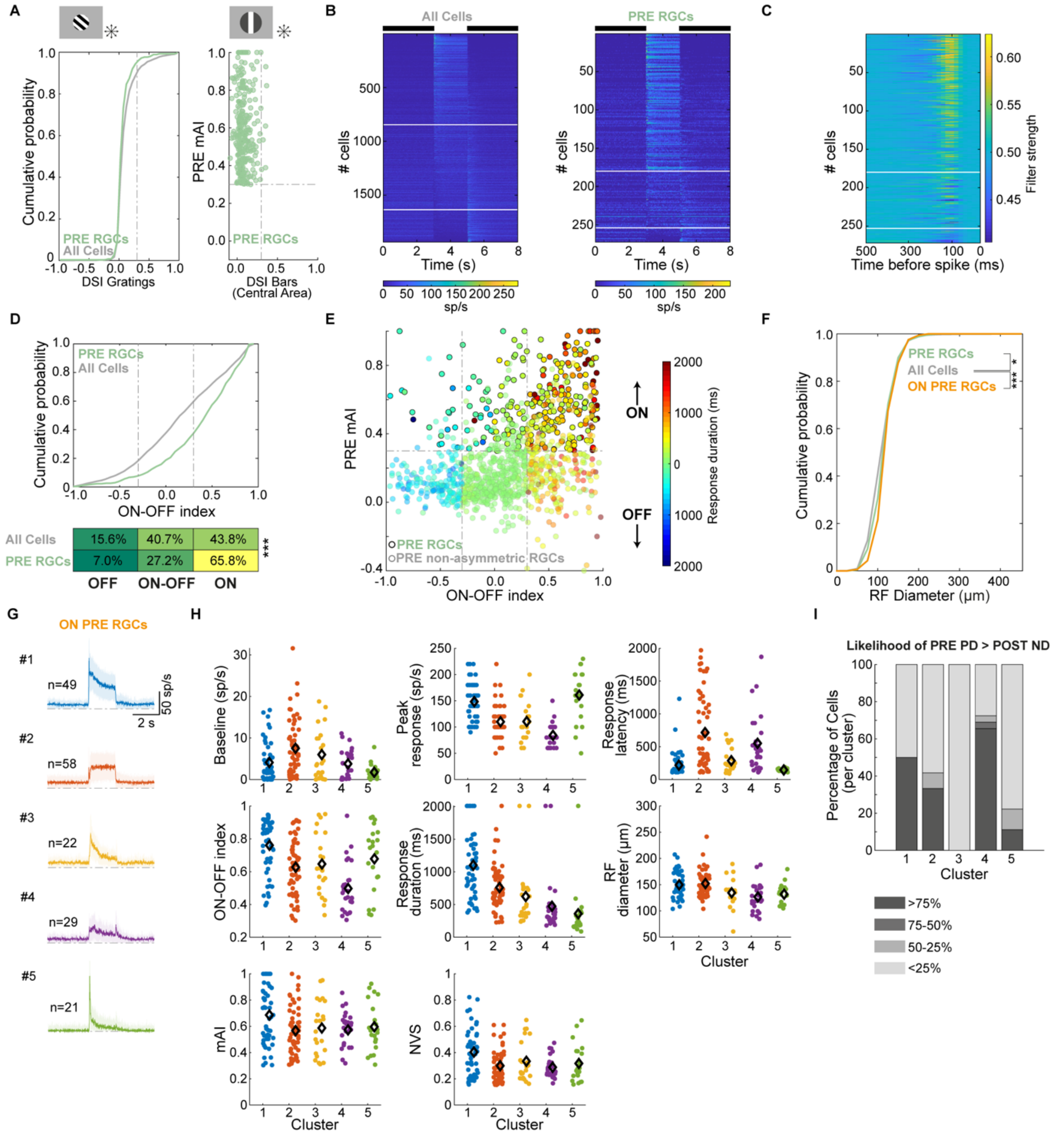

Fig. S5. Asymmetric PRE responses are observed in non-DS ON sustained RGCs, related to Fig. 1. **A.** Left: DSI distribution calculated in response to moving gratings in all tested RGCs (gray, 1850 RGCs in total, 15 experiments) and PRE RGCs (green, 244 RGCs). Only 11 (4.5%) of the 243 PRE RGCs were categorized as DSGCs in response to moving gratings, compared to 158 (8.5%) of the 1850 RGCs in total ($p<0.05$, Fisher's exact test). Right: mAI of the PRE response *vs.* DSI calculated in response to moving bars in the Central area. Only 1.8% of PRE RGCs are direction-selective to the moving bar in the Central area. Stimuli are illustrated on top. The dashed gray lines indicate DSI/mAI=0.3. **B.** Heatmaps showing responses to a full-field spot stimulus (indicated above) of all RGCs (left, 1935 RGCs, not presenting asymmetric PRE responses) and PRE RGCs (right, 272 RGCs), sorted according to their ON-OFF index (OFF, OOI≤-0.3; ON, OOI≥0.3; ON-OFF, -0.3<OOI<0.3). **C.** Temporal STA to the white-noise stimulus of PRE RGCs sorted as in B, right. The time below indicates the time before the spike. **D.** Top: ON-OFF index distributions calculated in response to the full-field spot stimulus of all RGCs (gray, 1935 RGCs, OOI=0.20±0.01) and PRE RGCs (green, 272 RGCs, OOI=0.41±0.03. Bottom: Percentages of ON, ON-OFF, and OFF RGCs in the two populations. χ^2 test. **E.** PRE mAI *vs.* OOI. Only RGCs where the number of spikes in the PRE response crossed a minimal

threshold per repetition are plotted (see Materials and Methods). Cells are color-coded according to the response duration, and PRE RGCs are encircled by black lines (PRE RGC: 272 out of 1185, 179 of them are ON cells, and their response duration is 743.3±37.5 ms). Of all 482 ON sustained RGCs, 108 showed an asymmetric PRE response. **F.** Mean RF diameter distributions for all RGCs (gray, 1770 RGCs, 135.0±0.8 μm), PRE RGCs (green, 250 RGCs, 139.4±1.9 μm), and ON PRE RGCs (orange, 163 out of 250 RGCs, 143.2±2.1 μm). Kruskal-Wallis test with Bonferroni post-hoc correction for multiple comparisons. **G.** Averaged PSTH (mean± SD) of ON PRE RGCs clusters in response to a full-filed spot stimulus (number of cells per cluster is indicated on the left). The dashed gray line defines zero activity. **H.** Distributions of response metrics across ON PRE RGCs clusters. Points are individual cells. Black diamonds represent the means for each metric. **I.** Percentage of cells grouped by the magnitude of the firing rate difference in PRE PD *vs*. POST ND after masking the Central area per cluster.

Data are reported as mean±SEM. *, **, ***: p<0.05, 0.01, 0.001, respectively.

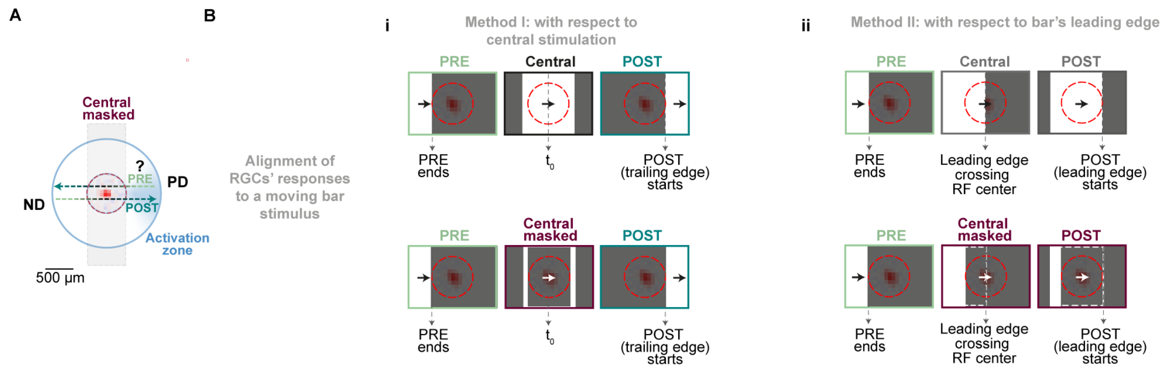

Fig. S6, related to Fig. 4. Alignment of RGC's responses to the leading/trailing edges of a moving bar stimulus and definition of the POST response in the masking experiments. A. The moving bar stimulus was repeated while masking the Central area to avoid desensitization and focus specifically on responses in the PRE preferred and POST null directions when the stimulus traverses the activation zone. Gray shades represent the masked area (width 700 µm). **B.** The location of the bar stimulus showed either over the full extent of the display (top) or masked by an occlude (bottom) for different time points. While in method **I**, the POST activity is defined as the response that starts when the trailing edge of the bar exits the Central area (i), in method **II**, the POST activity is calculated, comparably to the PRE response, as the response evoked by the leading edge of the moving bar (ii). Note that the calculation of the POST response in method **I** does not include the response to the bar's leading edge. This exclusion is necessary as it overlaps with the trailing edge within the classical receptive field. The central mask enables the isolation and assessment of the POST response to the bar's leading edge (method **II**, bottom).

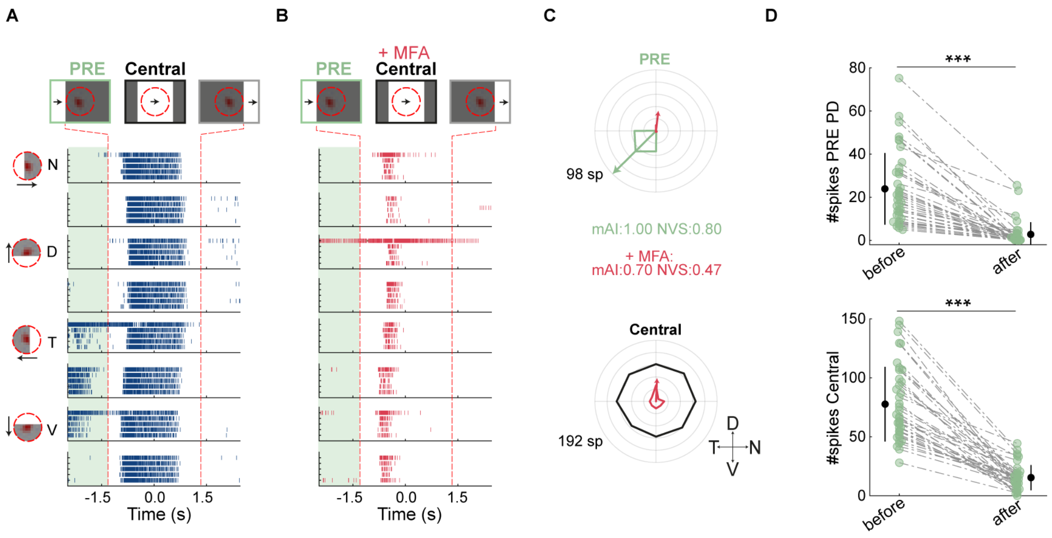

Fig. S7, related to Fig. 6. Pharmacological manipulations reveal that the extraclassical RF also relies on gap junctions. A, B. Raster plots of an example cell to bars moving in 8 different directions before (A) and after (B) MFA application. Notations as in Fig. 6A, B. **C.** Polar plots of the RGC response calculated in the PRE (top) and Central (bottom) responses. The mean response is plotted before (light green/black) and after (coral) MFA application. Notation as in Fig. 6C. **D.** Spike count during stimulus presentation in the PRE (top; before and after drug administration 23.91±2.66 and 2.81±0.90 spikes in the PRE preferred direction, respectively; mean±SEM) and Central area (bottom; before and after drug administration 77.64±5.07 and 15.38±1.71 spikes, respectively; mean±SEM) before and after MFA application. Mean±STD is shown for both axes, black lines. 39 cells, 3 experiments.
(D) *** p<0.001 according to the two-sided Wilcoxon signed-rank test.

SI References

1. W. Wei, J. Elstrott, M. B. Feller, Two-photon targeted recording of GFP-expressing neurons for light responses and live-cell imaging in the mouse retina. *Nat. Protoc.* **5**, 1347–1352 (2010).

2. K. P. Szatko, *et al.*, Neural circuits in the mouse retina support color vision in the upper visual field. *BioRxiv* (2019) https:/doi.org/10.1101/745539.

3. D. Karamanlis, T. Gollisch, Nonlinear spatial integration underlies the diversity of retinal ganglion cell responses to natural images. *J. Neurosci.* **41**, 3479–3498 (2021).

4. R. A. Warwick, A. S. Heukamp, S. Riccitelli, M. Rivlin-Etzion, Dopamine differentially affects retinal circuits to shape the retinal code. *J Physiol (Lond)* **601**, 1265–1286 (2023).

5. R. A. Warwick, *et al.*, Top-down modulation of the retinal code via histaminergic neurons of the hypothalamus. *Sci. Adv.* **10**, eadk4062 (2024).

6. L. Ankri, S. Riccitelli, M. Rivlin-Etzion, A new role for excitation in the retinal direction-selective circuit. *J Physiol (Lond)* (2024) https:/doi.org/10.1113/JP286581.

7. M. Pachitariu, N. A. Steinmetz, J. Colonell, Kilosort2 (2018) (August 8, 2021).

8. C. Rossant, K. D. Harris, Hardware-accelerated interactive data visualization for neuroscience in Python. *Front. Neuroinformatics* **7**, 36 (2013).

9. C. Rossant, *et al.*, Spike sorting for large, dense electrode arrays. *Nat. Neurosci.* **19**, 634–641 (2016).

10. R. Segev, J. Goodhouse, J. Puchalla, M. J. Berry, Recording spikes from a large fraction of the ganglion cells in a retinal patch. *Nat. Neurosci.* **7**, 1154–1161 (2004).

11. J. J. Jun, *et al.*, Fully integrated silicon probes for high-density recording of neural activity. *Nature* **551**, 232–236 (2017).

12. C. Stringer, *et al.*, Spontaneous behaviors drive multidimensional, brainwide activity. *Science* **364**, 255 (2019).

13. G. Nair, *et al.*, Effects of common anesthetics on eye movement and electroretinogram. *Doc. Ophthalmol.* **122**, 163–176 (2011).

14. J. Schindelin, *et al.*, Fiji: an open-source platform for biological-image analysis. *Nat. Methods* **9**, 676–682 (2012).

15. P. Shamash, M. Carandini, K. D. Harris, N. A. Steinmetz, A tool for analyzing electrode tracks from slice histology. *BioRxiv* (2018) https:/doi.org/10.1101/447995.

16. R. A. Warwick, N. Kaushansky, N. Sarid, A. Golan, M. Rivlin-Etzion, Inhomogeneous encoding of the visual field in the mouse retina. *Curr. Biol.* **28**, 655-665.e3 (2018).

17. B. Krieger, M. Qiao, D. L. Rousso, J. R. Sanes, M. Meister, Four alpha ganglion cell types in mouse retina: Function, structure, and molecular signatures. *PLoS ONE* **12**, e0180091 (2017).

18. W. Sun, N. Li, S. He, Large-scale morphological survey of mouse retinal ganglion cells. *J. Comp. Neurol.* **451**, 115–126 (2002).

19. A. Bleckert, G. W. Schwartz, M. H. Turner, F. Rieke, R. O. L. Wong, Visual space is represented by nonmatching topographies of distinct mouse retinal ganglion cell types. *Curr. Biol.* **24**, 310–315 (2014).

20. J. A. Bae, *et al.*, Digital Museum of Retinal Ganglion Cells with Dense Anatomy and Physiology. *Cell* **173**, 1293-1306.e19 (2018).

21. J. Goetz, *et al.*, Unified classification of mouse retinal ganglion cells using function, morphology, and gene expression. *Cell Rep.* **40**, 111040 (2022).

22. P. Berens, circstat : a *MATLAB* toolbox for circular statistics. *J. Stat. Softw.* **31** (2009).
